# Supplementary material for: Vaccination decreases the risk of influenza A virus reassortment but not genetic variation in pigs
Source: eLife. 2022 Sep 2;11:e78618. doi: 10.7554/eLife.78618 (PMC9439680; doi:10.7554/eLife.78618)
Supplement: Figure 2—source data 1. — Phylogenetic trees of eight influenza A virus (IAV) genes from viral plaque isolates were constructed using the maximum likelihood method with 1000 bootstraps and best-fitted nucleotide substitution model. The color of leaf nodes indicates the genetic origin of the IAV segments. The red and green taxa represent the sequences are derived from the H1N1 and H3N2 challenge viruses, respectively. The blue taxa are sequences that originated from the mixed genotype plaques. The genomes of H1N1 and H3N2 challenge viruses are displayed as pink taxa, and the other reference sequences are shown as black taxa. The bootstrap values are proportionally displayed by the circle size of the tree nodes. [file elife-78618-fig2-data1.pdf]

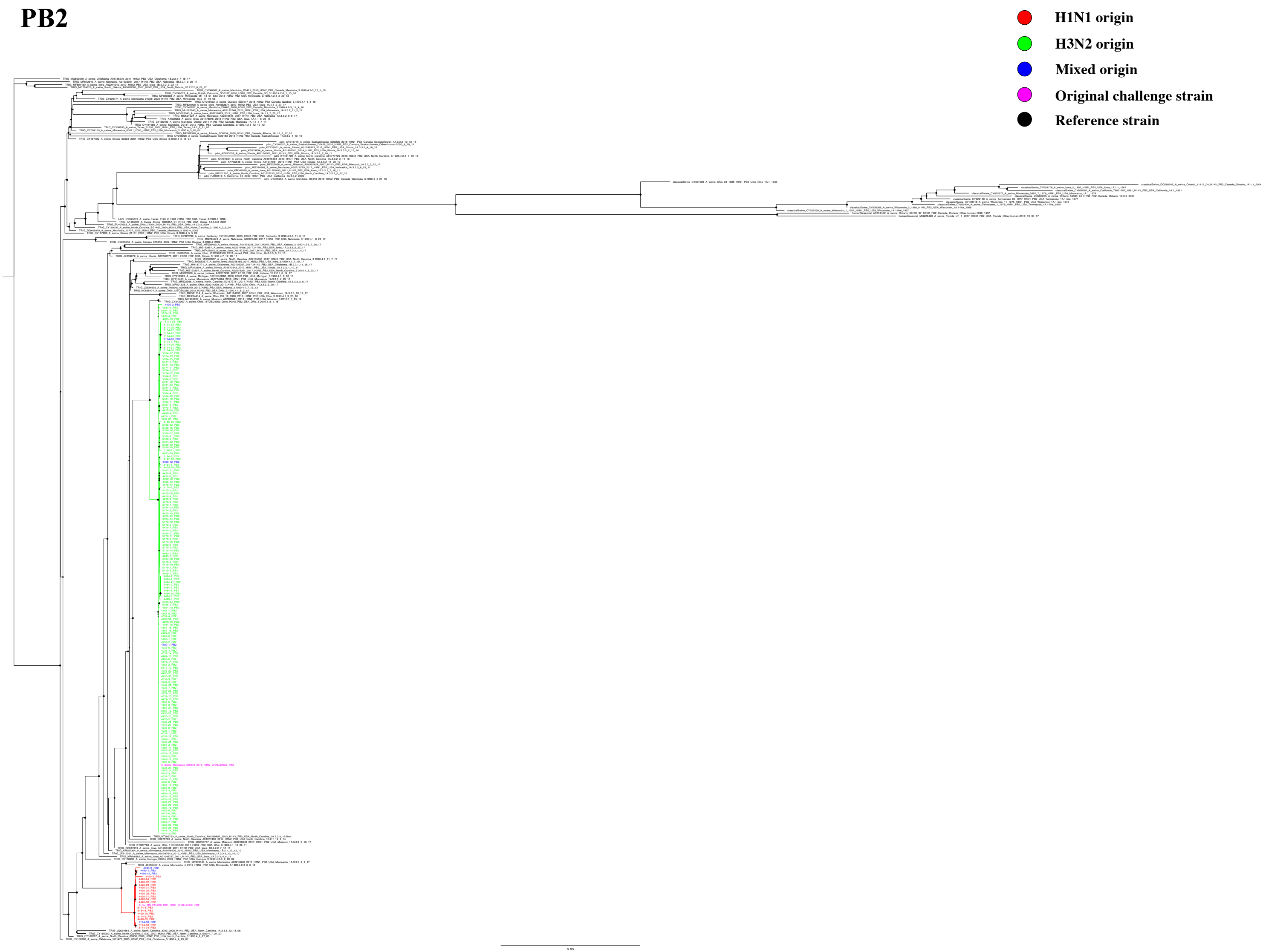
PB2
H1N1 origin
H3N2 origin
Mixed origin
Original challenge strain
Reference strain
0.05

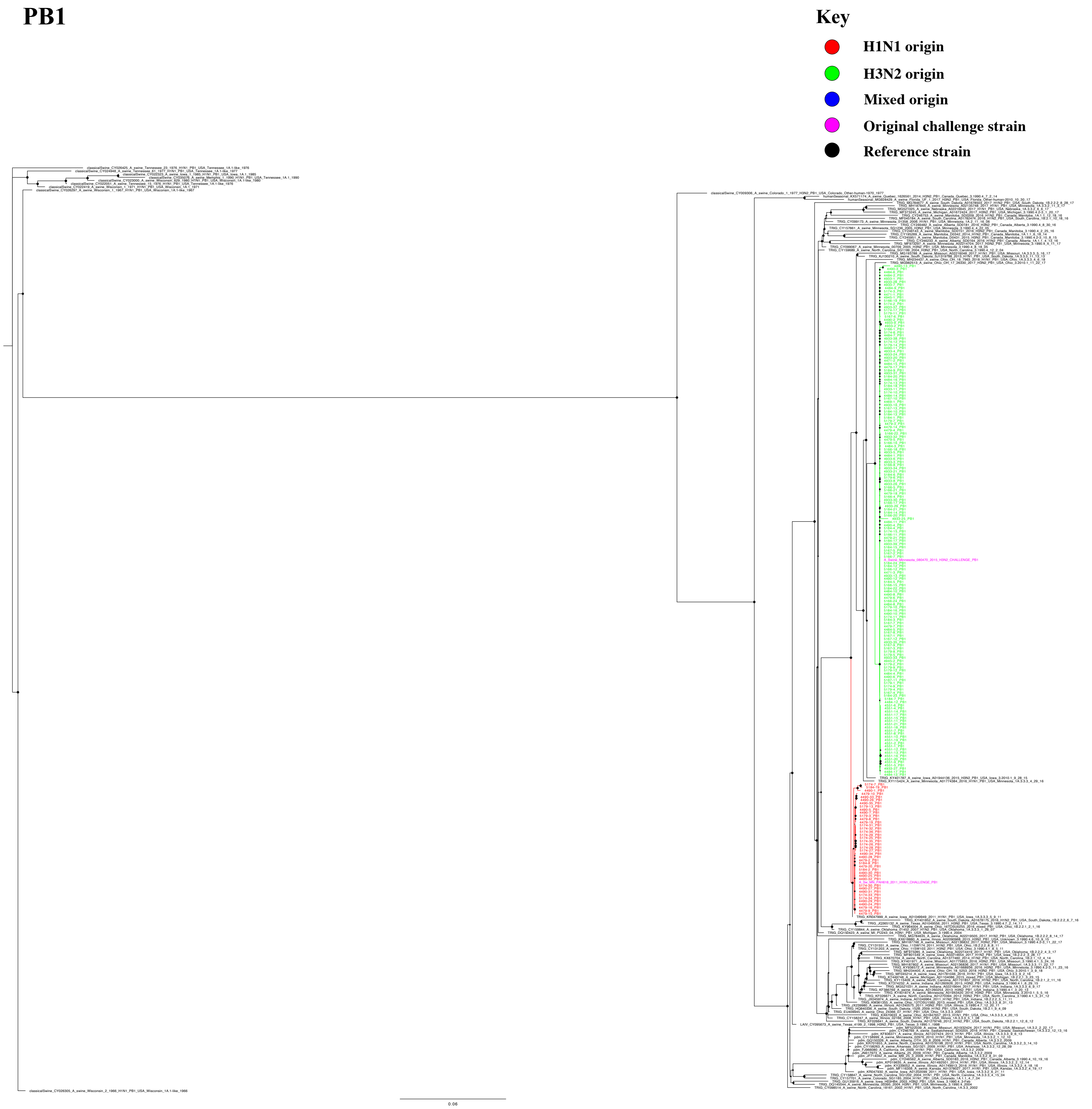
PB1
Key
H1N1 origin
H3N2 origin
Mixed origin
Original challenge strain
Reference strain
0.06

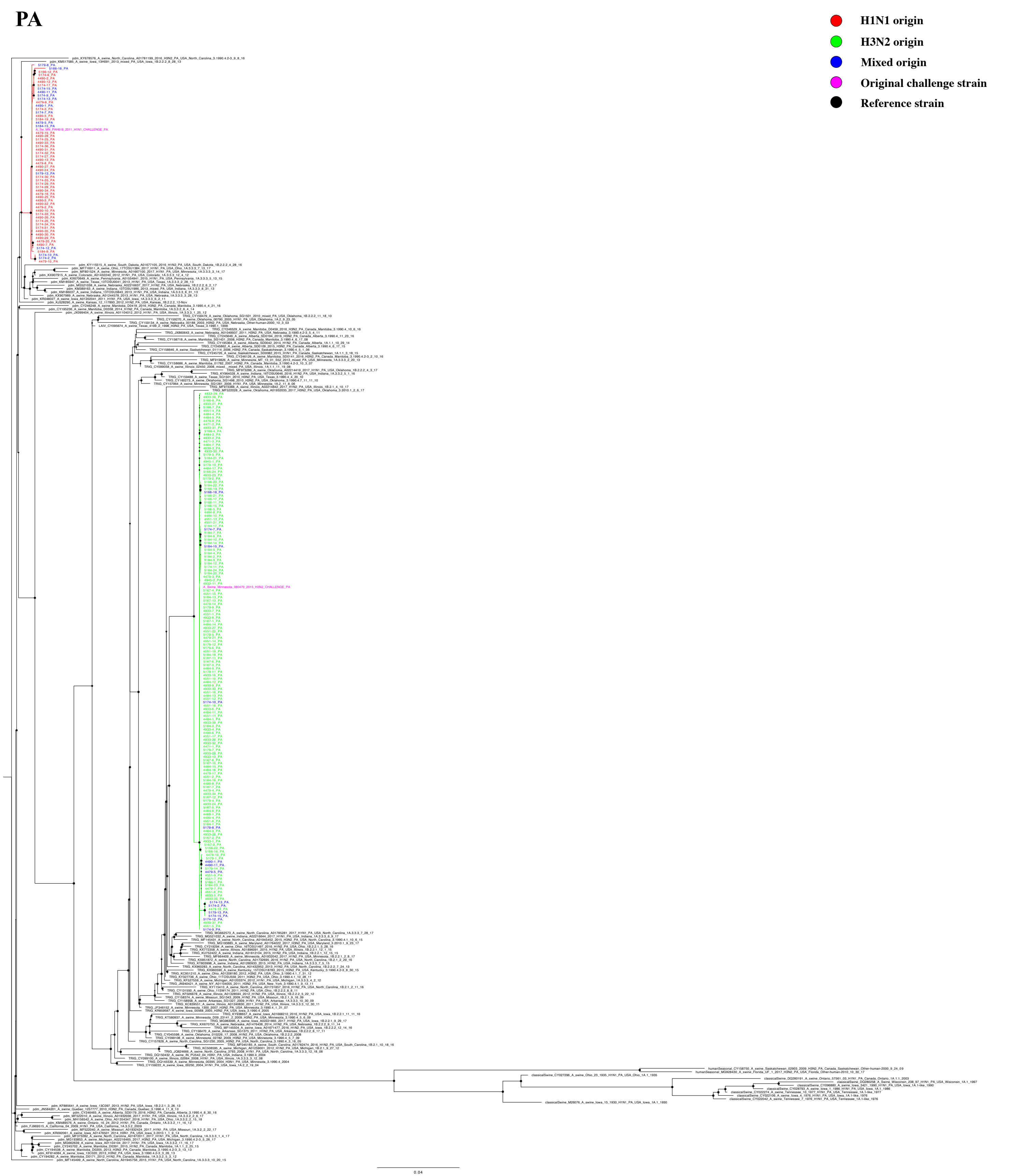
PA
H1N1 origin
H3N2 origin
Mixed origin
Original challenge strain
Reference strain
0.04

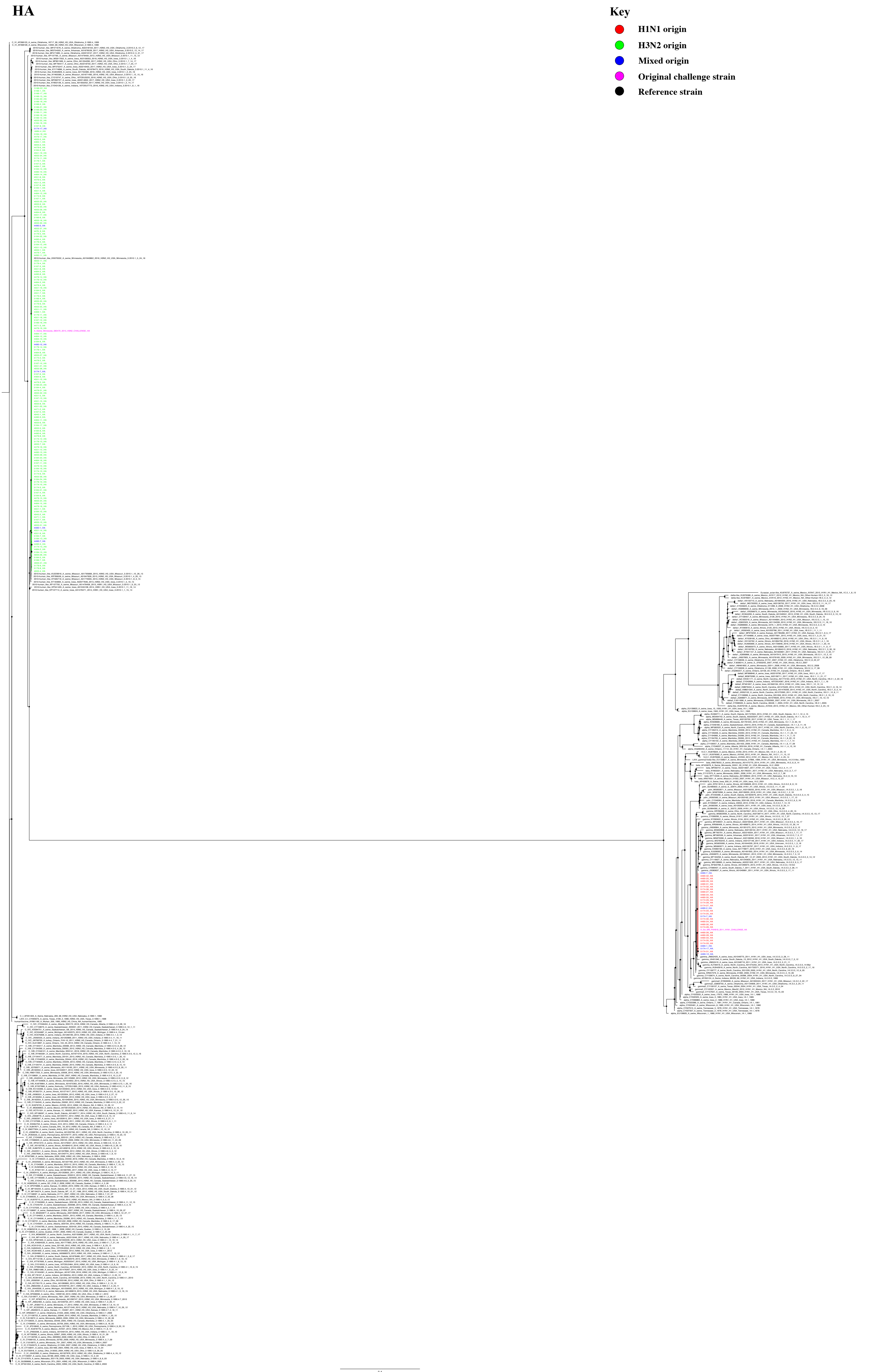
HA
Key
H1N1 origin
H3N2 origin
Mixed origin
Original challenge strain
Reference strain
0.4

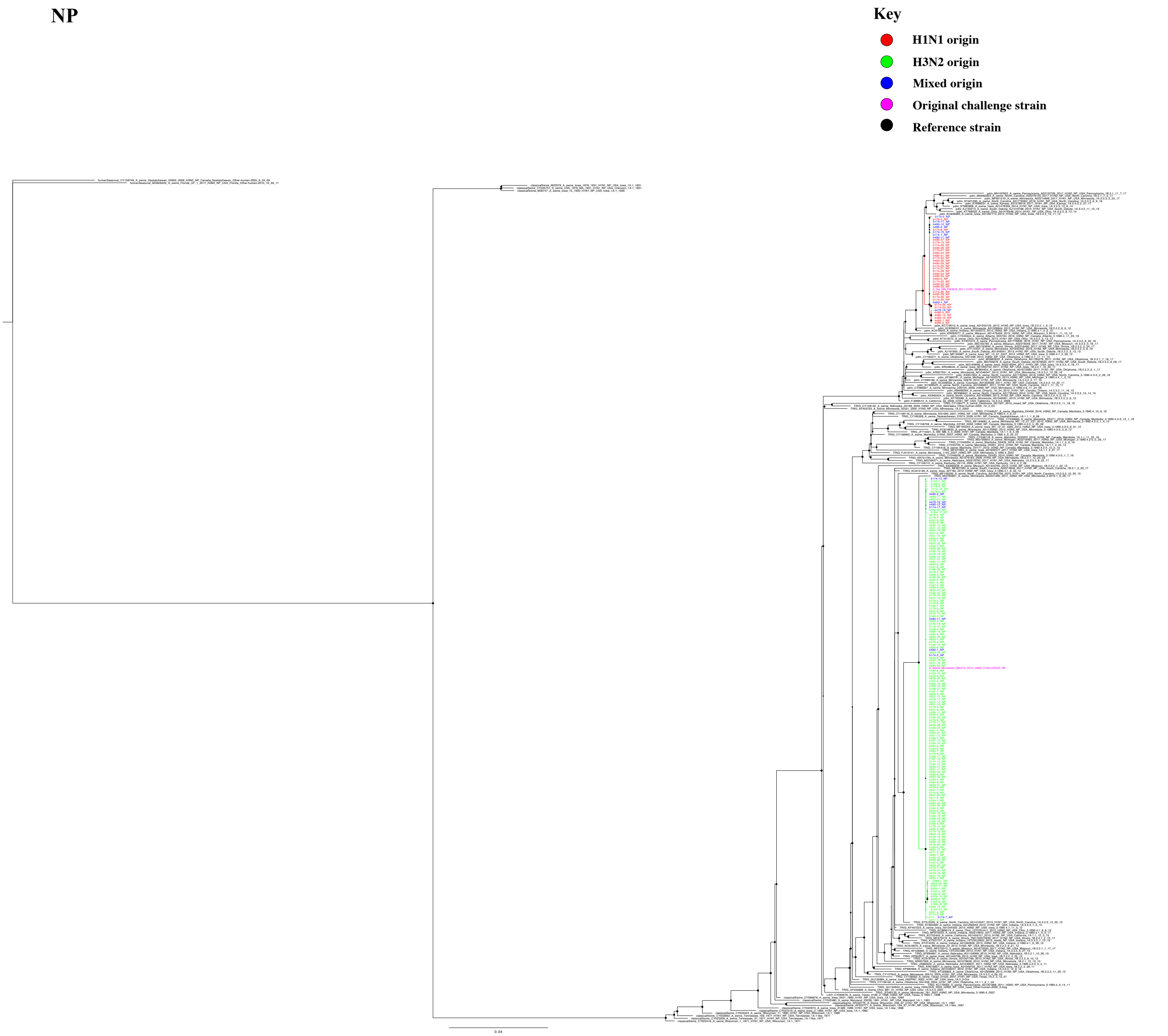
NP
Key
H1N1 origin
H3N2 origin
Mixed origin
Original challenge strain
Reference strain
0.04

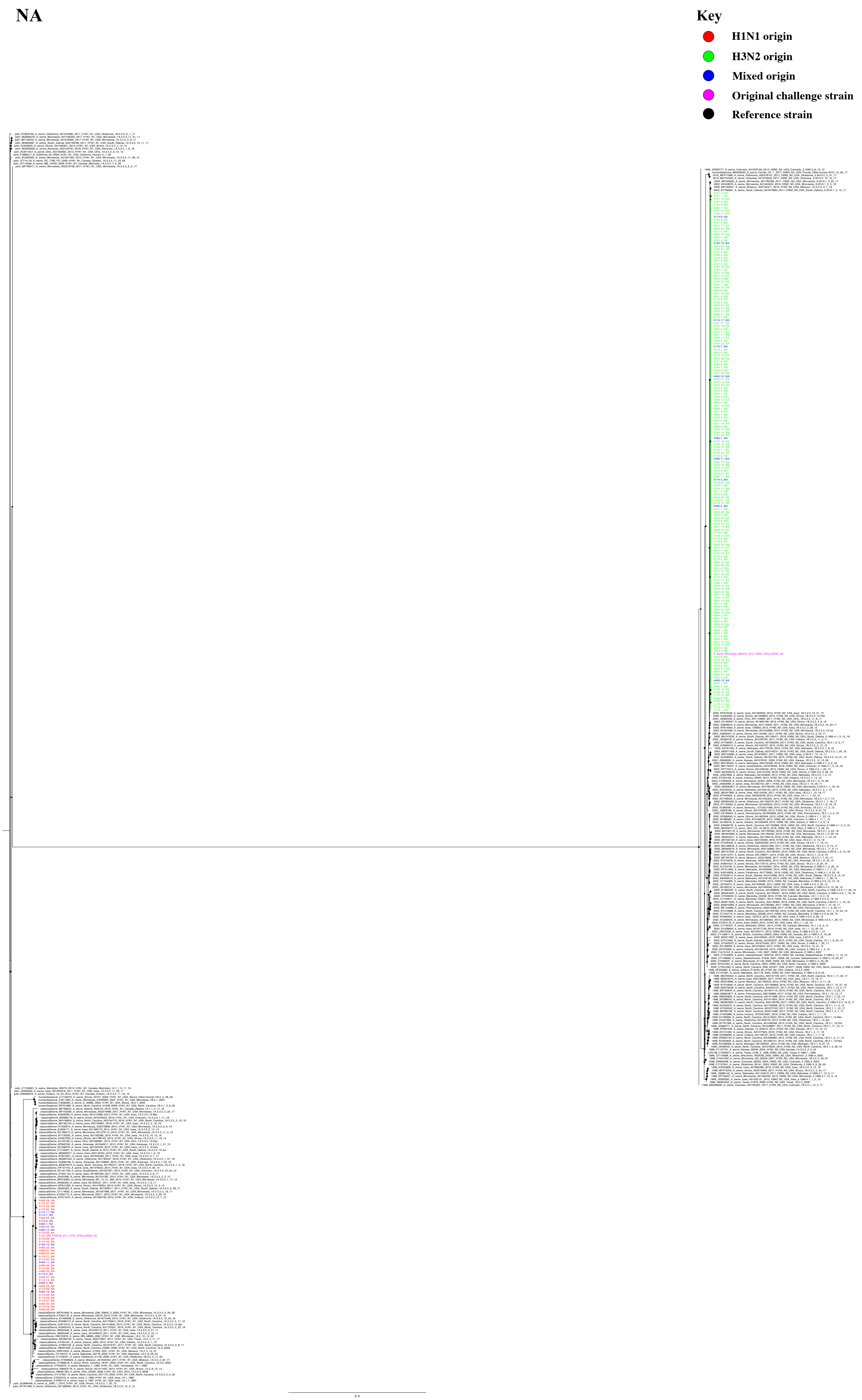
NA
Key
H1N1 origin
H3N2 origin
Mixed origin
Original challenge strain
Reference strain
2.0

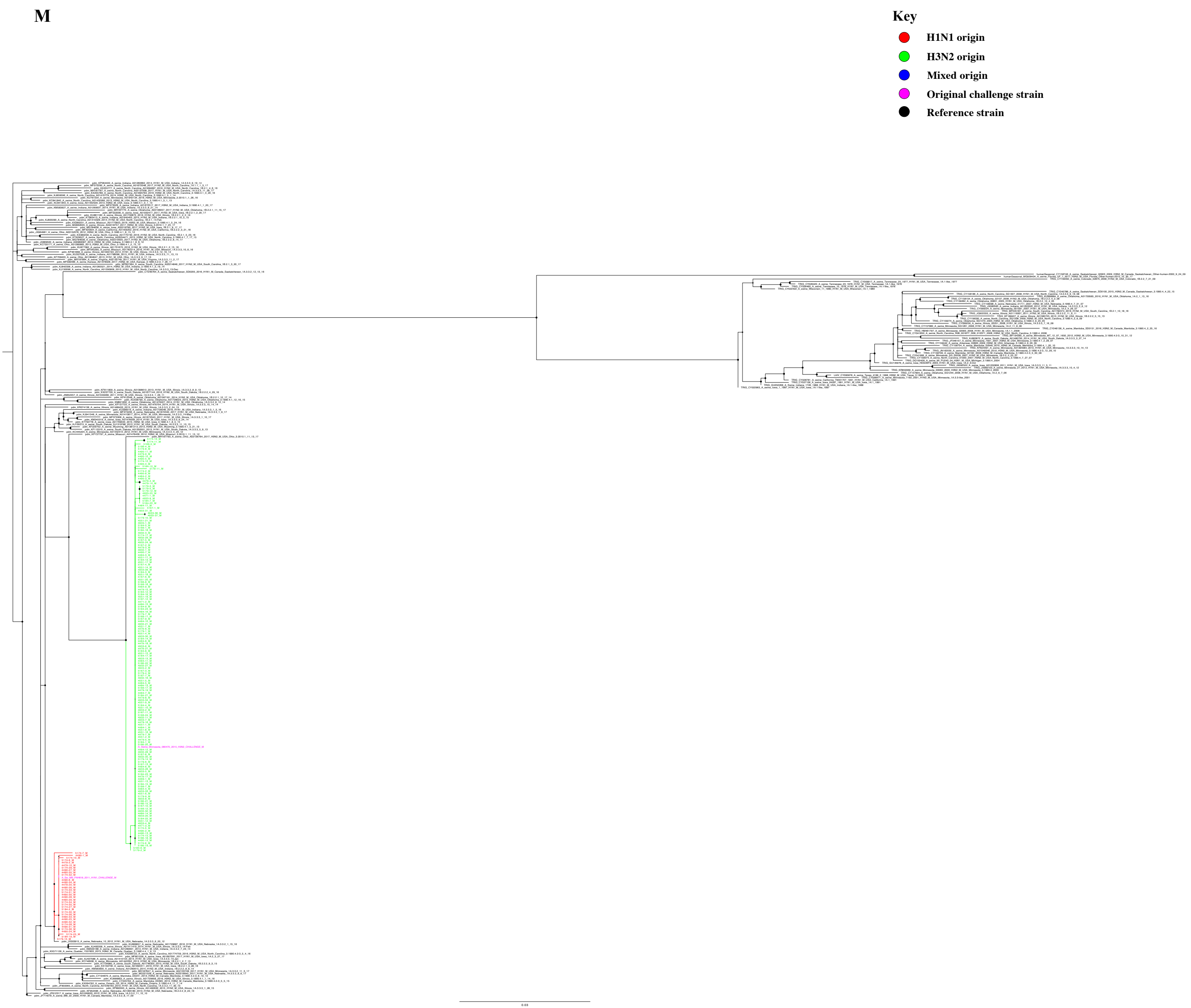
M
Key
H1N1 origin
H3N2 origin
Mixed origin
Original challenge strain
Reference strain
0.03

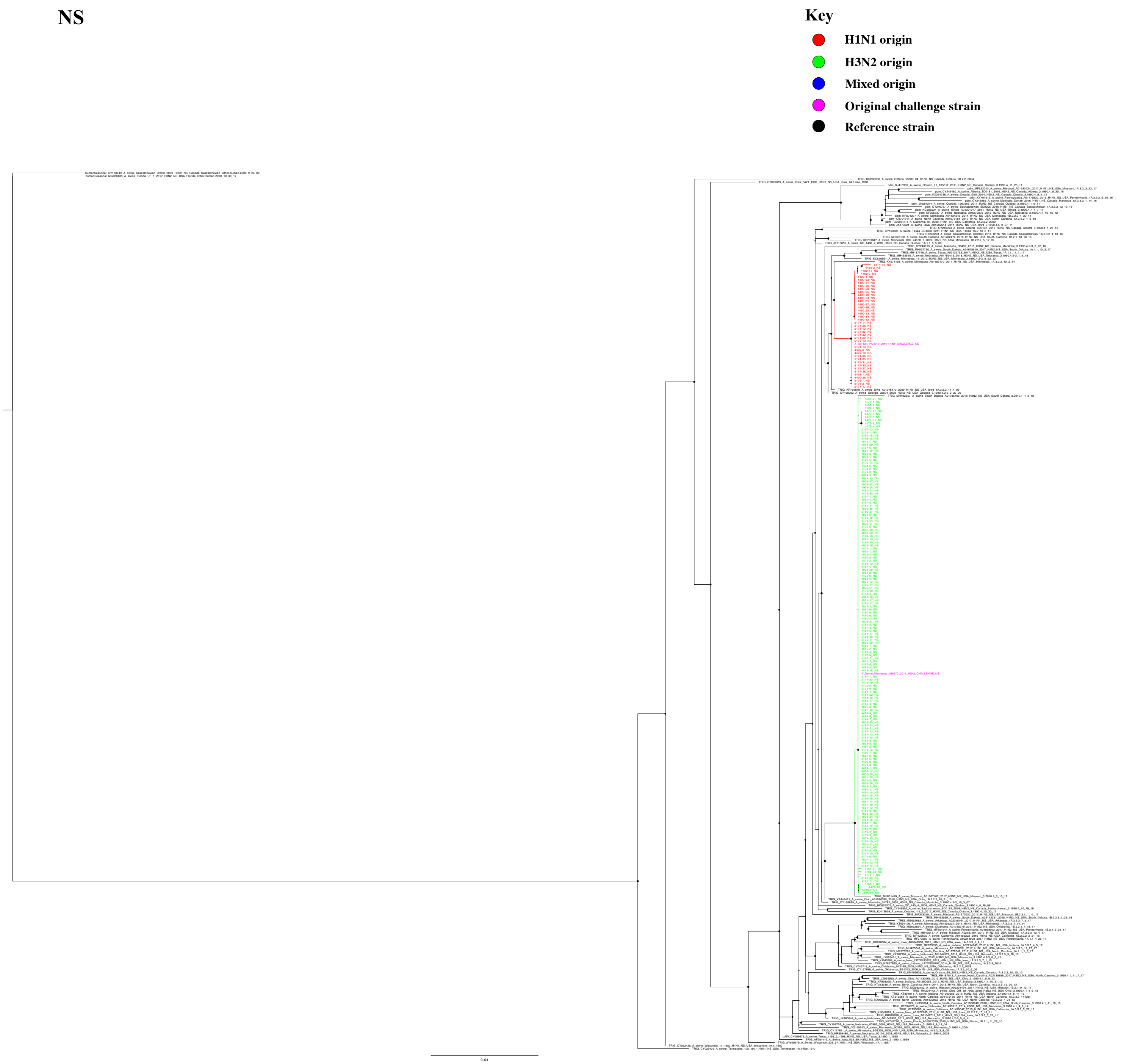
NS
Key
H1N1 origin
H3N2 origin
Mixed origin
Original challenge strain
Reference strain
0.04
