# Supplementary material for: Vaccination decreases the risk of influenza A virus reassortment but not genetic variation in pigs
Source: eLife. 2022 Sep 2;11:e78618. doi: 10.7554/eLife.78618 (PMC9439680; doi:10.7554/eLife.78618)
Supplement: Figure 2—source data 3. [file elife-78618-fig2-data3.docx]

| **Genotype** | **PB2** | **PB1** | **PA** | **HA** | **NP** | **NA** | **M** | **NS** | **Reassortant** | **Total number** |
| --- | --- | --- | --- | --- | --- | --- | --- | --- | --- | --- |
| **H3N2** |  |  |  |  |  |  |  |  | **No** | **137** |
| **H1N1** |  |  |  |  |  |  |  |  | **No** | **11** |
| **R01** |  |  |  |  |  |  |  |  | **Yes** | **3** |
| **R02** |  |  |  |  |  |  |  |  | **Yes** | **2** |
| **R03** |  |  |  |  |  |  |  |  | **Yes** | **4** |
| **R04** |  |  |  |  |  |  |  |  | **Yes** | **1** |
| **R05** |  |  |  |  |  |  |  |  | **Yes** | **1** |
| **R06** |  |  |  |  |  |  |  |  | **Yes** | **1** |
| **R07** |  |  |  |  |  |  |  |  | **Yes** | **1** |
| **R08** |  |  |  |  |  |  |  |  | **Yes** | **1** |
| **R09** |  |  |  |  |  |  |  |  | **Yes** | **1** |
| **R10** |  |  |  |  |  |  |  |  | **Yes** | **2** |
| **R11** |  |  |  |  |  |  |  |  | **Yes** | **1** |
| **R12** |  |  |  |  |  |  |  |  | **Yes** | **1** |
| **R13** |  |  |  |  |  |  |  |  | **Yes** | **9** |
| **R14** |  |  |  |  |  |  |  |  | **Yes** | **2** |
| **R15** |  |  |  |  |  |  |  |  | **Yes** | **1** |
| **R16** |  |  |  |  |  |  |  |  | **Yes** | **1** |
| **R17** |  |  |  |  |  |  |  |  | **Yes** | **1** |
| **M01** |  |  |  |  |  |  |  |  | **Yes** | **3** |
| **M02** |  |  |  |  |  |  |  |  | **Yes** | **1** |
| **M03** |  |  |  |  |  |  |  |  | **Yes** | **1** |
| **M04** |  |  |  |  |  |  |  |  | **Yes** | **2** |
| **M05** |  |  |  |  |  |  |  |  | **Yes** | **1** |
| **M06** |  |  |  |  |  |  |  |  | **Yes** | **2** |
| **M07** |  |  |  |  |  |  |  |  | **Yes** | **1** |
| **M08** |  |  |  |  |  |  |  |  | **Yes** | **1** |
| **M09** |  |  |  |  |  |  |  |  | **Yes** | **1** |
| **M10** |  |  |  |  |  |  |  |  | **Yes** | **1** |
| **M11** |  |  |  |  |  |  |  |  | **Yes** | **2** |
| **M12** |  |  |  |  |  |  |  |  | **Yes** | **1** |
| **M13** |  |  |  |  |  |  |  |  | **Yes** | **1** |
| **M14** |  |  |  |  |  |  |  |  | **Yes** | **1** |
| **M15** |  |  |  |  |  |  |  |  | **Yes** | **1** |
| **M16** |  |  |  |  |  |  |  |  | **Yes** | **1** |
| **Total** |  | | | | | | | | | **202** |
