# Supplementary material for: Vaccination decreases the risk of influenza A virus reassortment but not genetic variation in pigs
Source: eLife. 2022 Sep 2;11:e78618. doi: 10.7554/eLife.78618 (PMC9439680; doi:10.7554/eLife.78618)
Supplement: Figure 3—source data 1. [file elife-78618-fig3-data1.docx]

**Figure 3 – source data 1. Infection dynamics of H1N1 and H3N2 challenge viruses assessed by subtype specific rRT-PCR in nasal swabs and broncho-alveolar fluid samples (BALF).**

| Pig ID | Treatment | Nasal Swabs (Ct)^a^ | | | | | | | | | | BALF (Ct) | | No. of days pigs  were co-infected^b^ | No. of reassortants/ total plaques isolated |
| --- | --- | --- | --- | --- | --- | --- | --- | --- | --- | --- | --- | --- | --- | --- | --- |
|  |  | 2 dpc^c^ | | 3 dpc | | 4 dpc | | 5 dpc | | 6 dpc | | 7 dpc | |  |  |
|  |  | H1 | H3 | H1 | H3 | H1 | H3 | H1 | H3 | H1 | H3 | H1 | H3 |  |  |
| 4471 | PRIME BOOST | 35.02 | Neg | Neg | Neg | Neg | 37.20 | Neg | 36.97 | Neg | Neg | Neg | 25.41 | 0 | 0/3 |
| 4551 | PRIME BOOST | Neg | Neg | Neg | Neg | Neg | 30.79 | Neg | 28.38 | Neg | 28.25 | Neg | 27.47 | 0 | 0/20 |
| 5184 | PRIME BOOST | Neg | Neg | Neg | Neg | Neg | Neg | Neg | Neg | Neg | 35.31 | 37.41 | 24.44 | 1 | 4/23 |
| 4469 | PRIME BOOST | Neg | Neg | Neg | Neg | Neg | Neg | Neg | Neg | Neg | Neg | Neg | 32.64 | 0 | 0/1 |
| 4933 | PRIME BOOST | Neg | Neg | Neg | 33.44 | Neg | 31.65 | Neg | 33.75 | Neg | 33.75 | Neg | 29.59 | 0 | 0/29 |
| 4479 | SINGLE LAIV | Neg | 31.69 | Neg | 31.50 | Neg | 29.95 | 28.37 | 26.47 | Neg | 32.89 | 31.16 | 31.13 | 2 | 11/17 |
| 4484 | SINGLE LAIV | Neg | 33.56 | Neg | 31.61 | Neg | 28.65 | 33.52 | 29.46 | Neg | 33.23 | Neg | 24.04 | 1 | 0/17 |
| 4945 | SINGLE LAIV | Neg | 33.79 | 35.98 | 29.98 | Neg | 26.25 | Neg | 29.26 | Neg | 33.62 | Neg | 28.29 | 1 | 0/2 |
| 5166 | SINGLE LAIV | Neg | 32.69 | Neg | 28.95 | Neg | 27.98 | Neg | 32.18 | Neg | 32.29 | 27.18 | 24.62 | 1 | 2/16 |
| 4490 | NO VAC | Neg | Neg | Neg | Neg | 29.04 | 28.32 | 22.38 | 23.89 | 28.78 | 26.16 | 22.81 | 24.83 | 4 | 11/24 |
| 5167 | NO VAC | Neg | 31.27 | Neg | 27.81 | Neg | 30.06 | Neg | 25.36 | 35.95 | 25.82 | 32.05 | 27.23 | 2 | 0/13 |
| 5174 | NO VAC | 34.48 | 34.49 | 32.99 | 32.79 | 31.50 | 30.70 | 27.71 | 22.86 | 33.55 | 25.34 | 25.97 | 22.27 | 6 | 23/23 |
| 5179 | NO VAC | Neg | 32.93 | Neg | 29.01 | Neg | 28.21 | Neg | 29.65 | Neg | 28.08 | 25.88 | 24.11 | 1 | 3/14 |

1. Ct: cycle threshold virus. Ct values below 39 were considered positive.
2. The number of days pigs were co-infected is defined as the number of days that both H1 and H3 IAV were detected in the samples collected from the pig nasal cavities or lungs by a HA subtype-specific multiplex real-time reverse transcription-polymerase chain reaction (RRT-PCR) test.
3. Dpc: days post contact.
