# Supplementary material for: Vaccination decreases the risk of influenza A virus reassortment but not genetic variation in pigs
Source: eLife. 2022 Sep 2;11:e78618. doi: 10.7554/eLife.78618 (PMC9439680; doi:10.7554/eLife.78618)
Supplement: Figure 5—source data 1. [file elife-78618-fig5-data1.docx]

**Figure 5 – source data 1. Values of nucleotide diversity in H1N1 and H3N2 challenge viruses by coding regions and treatment groups.**

| **Gene** | **Infection** | **Treatment** | **Nucleotide diversity** | | | |
| --- | --- | --- | --- | --- | --- | --- |
|  |  |  | **piN**  **(Standard deviation)** | **piS**  **(Standard deviation)** | **piN/piS** | **P value** |
| Whole-genome level | H1N1 | PRIME BOOST | 1.422982e-04 (9.405784e-05) | 0.0002172992 (0.0001456324) | 0.6548492 | 0.291 |
|  |  | SINGLE LAIV | 4.882586e-05 (3.063717e-05) | 0.0002849059 (0.0001221796) | 0.1713754 | 0.001 |
|  |  | NO VAC | 8.317157e-05 (7.544864e-05) | 0.0002635756 (0.0001471726) | 0.3155511 | 0.036 |
|  | H3N2 | PRIME BOOST | 8.678509e-05 (9.891502e-05) | 0.0002621745 (2.344853e-04) | 0.3310203 | 0.040 |
|  |  | SINGLE LAIV | 3.942700e-05 (4.455551e-05) | 0.0002997717 (1.499483e-04) | 0.1315234 | 0.014 |
|  |  | NO VAC | 4.730000e-05 (3.554274e-05) | 0.0002900643 (7.503045e-05) | 0.1630673 | 0.007 |
| PB2 | H1N1 | PRIME BOOST | 2.576880e-04 (2.903757e-04) | 0.0005142157 (0.0006515918) | 0.5011282 | 0.638 |
|  |  | SINGLE LAIV | 2.355714e-05 (3.027897e-05) | 0.0003564546 (0.0002861042) | 0.06608735 | 0.021 |
|  |  | NO VAC | 6.533757e-05 (1.076469e-04) | 0.0002242704 (0.0002283647) | 0.2913339 | 0.047 |
|  | H3N2 | PRIME BOOST | 1.118274e-04 (1.641121e-04) | 0.0005652066 (0.0005909784) | 0.1978523 | 0.036 |
|  |  | SINGLE LAIV | 4.786233e-05 (6.083782e-05) | 0.0005848542 (0.0003471910) | 0.08183634 | 0.014 |
|  |  | NO VAC | 7.203050e-05 (6.584911e-05) | 0.0010220865 (0.0006506746) | 0.07047398 | 0.064 |
| PB1 | H1N1 | PRIME BOOST | 1.963260e-04 (9.701364e-05) | 0.000280117 (0.0003844214) | 0.7008714 | 0.751 |
|  |  | SINGLE LAIV | 2.207143e-05  (3.468154e-05) | 0.0003388680  (0.0003782360) | 0.06513282 | 0.068 |
|  |  | NO VAC | 9.470057e-05  (1.267962e-04) | 0.0004252200  (0.0002372126) | 0.2227096 | 0.036 |
|  | H3N2 | PRIME BOOST | 0.0001409929  (1.560628e-04) | 0.0001689430  (0.0002684783) | 0.834559 | 0.775 |
|  |  | SINGLE LAIV | 0.0000758445  (1.080137e-04) | 0.0002455283 (0.0003233749) | 0.3089033 | 0.211 |
|  |  | NO VAC | 0.0000219000  (3.469870e-06) | 0.0001406860  (0.0001455557) | 0.1556658 | 0.294 |
| PB1-F2 | H1N1 | PRIME BOOST | 1.169138e-03  (1.653411e-03) | 0.000000e+00  (0.000000e+00) | NA | 0.500 |
|  |  | SINGLE LAIV | 3.458244e-04  (7.983380e-04) | 0.000000e+00  (0.000000e+00) | NA | 0.295 |
|  |  | NO VAC | 3.159671e-05  (8.359705e-05) | 7.474271e-05  (0.0001977506) | 0.4227397 | 0.356 |
|  | H3N2 | PRIME BOOST | 2.855567e-05  (0.0000856670) | 0.000000e+00  (0.000000e+00) | NA | 0.347 |
|  |  | SINGLE LAIV | 0.000000e+00  (0.000000e+00) | 0.000000e+00  (0.000000e+00) | NA | NA |
|  |  | NO VAC | 0.000000e+00  (0.000000e+00) | 0.000000e+00  (0.000000e+00) | NA | NA |
| PA | H1N1 | PRIME BOOST | 7.175425e-05  (7.798960e-05) | 0.0000333605  (0.0000667210) | 2.150875 | 0.567 |
|  |  | SINGLE LAIV | 4.721743e-05  (5.077415e-05) | 0.0002136707  (0.0002291208) | 0.2209822 | 0.063 |
|  |  | NO VAC | 7.100843e-05  (1.044903e-04) | 0.0003701989  (0.0005256765) | 0.1918116 | 0.218 |
|  | H3N2 | PRIME BOOST | 9.345875e-05  (1.163594e-04) | 0.0001689700  (0.0003615380) | 0.5531085 | 0.618 |
|  |  | SINGLE LAIV | 3.952233e-05 (6.319568e-05) | 0.000236675 (0.0004762967) | 0.1669899 | 0.376 |
|  |  | NO VAC | 3.292500e-05  (3.148983e-05) | 0.0001543330  (0.0001683670) | 0.2133374 | 0.275 |
| PA-X | H1N1 | PRIME BOOST | 8.898050e-05  (1.449145e-04) | 0.000000e+00  (0.000000e+00) | NA | 0.307 |
|  |  | SINGLE LAIV | 9.368986e-05  (1.256255e-04) | 0.0001155797  (0.0001771518) | 0.8106083 | 0.775 |
|  |  | NO VAC | 4.443471e-05  (4.965662e-05) | 0.0004701817  (0.0011736942) | 0.0945054 | 0.381 |
|  | H3N2 | PRIME BOOST | 1.438287e-04  (0.0002448067) | 2.080838e-05  (5.885497e-05) | 6.912057 | 0.231 |
|  |  | SINGLE LAIV | 2.823450e-05 (6.916012e-05) | 2.790500e-05 (6.835301e-05) | 1.011808 | 0.994 |
|  |  | NO VAC | 4.033575e-05  (0.0000806715) | 9.797800e-05  (1.959560e-04) | 0.4116817 | 0.658 |
| HA | H1N1 | PRIME BOOST | 1.112333e-04  (1.583274e-04) | 0.0005233233 (0.0004716215) | 0.2125518 | 0.184 |
|  |  | SINGLE LAIV | 7.207143e-05  (1.026763e-04) | 0.0006752857  (0.0007604864) | 0.1067273 | 0.058 |
|  |  | NO VAC | 8.725714e-05  (9.777048e-05) | 0.0001408143  (0.0003068438) | 0.6196611 | 0.678 |
|  | H3N2 | PRIME BOOST | 2.135100e-05  (2.357706e-05) | 0.0001247720  (0.0002913994) | 0.1711201 | 0.291 |
|  |  | SINGLE LAIV | 3.124167e-05  (5.829104e-05) | 0.0003777733  (0.0005021299) | 0.08269952 | 0.172 |
|  |  | NO VAC | 1.469750e-05  (1.855108e-05) | 0.0000703975  (0.0001060913) | 0.2087787 | 0.421 |
| NP | H1N1 | PRIME BOOST | 3.133525e-04  (3.546176e-04) | 6.017900e-05  (8.510596e-05) | 5.207007 | 0.565 |
|  |  | SINGLE LAIV | 2.971429e-06  (7.861661e-06) | 1.255714e-05  (3.322308e-05) | 0.2366326 | 0.501 |
|  |  | NO VAC | 6.088400e-05  (8.886361e-05) | 3.682129e-05  (6.406592e-05) | 1.6535 | 0.593 |
|  | H3N2 | PRIME BOOST | 9.741173e-05  (1.478800e-04) | 0.0002438515  (4.436895e-04) | 0.3994715 | 0.158 |
|  |  | SINGLE LAIV | 9.816667e-06  (1.607811e-05) | 0.0004303302  (6.704243e-04) | 0.02281194 | 0.178 |
|  |  | NO VAC | 2.215000e-05  (2.574497e-05) | 0.0000575350  (7.708043e-05) | 0.3849831 | 0.491 |
| NA | H1N1 | PRIME BOOST | 2.304310e-04  (NA) | 0.0002746330  (NA) | 0.8390507 | NA |
|  |  | SINGLE LAIV | 5.417657e-05  (8.231250e-05) | 0.0003113750  (0.0005705153) | 0.1739914 | 0.297 |
|  |  | NO VAC | 1.775523e-04  (2.081088e-04) | 0.0002248094  (0.0002631608) | 0.7897904 | 0.685 |
|  | H3N2 | PRIME BOOST | 5.021100e-05  (3.885610e-05) | 9.988945e-05  (0.0001794232) | 0.5026657 | 0.365 |
|  |  | SINGLE LAIV | 4.057817e-05  (4.845540e-05) | 2.510172e-04  (0.0002776949) | 0.1616549 | 0.079 |
|  |  | NO VAC | 1.344940e-04  (2.328601e-04) | 4.750387e-04  (0.0008009431) | 0.2831222 | 0.321 |
| M1 | H1N1 | PRIME BOOST | 1.906183e-04  (0.0001178895) | 3.168367e-04  (3.679739e-04) | 0.6016295 | 0.672 |
|  |  | SINGLE LAIV | 9.054886e-05  (0.0001251853) | 3.672429e-05  (6.299647e-05) | 2.465639 | 0.379 |
|  |  | NO VAC | 1.210221e-04  (0.0002338880) | 8.904243e-05  (1.828629e-04) | 1.359151 | 0.768 |
|  | H3N2 | PRIME BOOST | 1.091210e-04  (2.676841e-04) | 1.253893e-04  (0.0002434758) | 0.8702577 | 0.869 |
|  |  | SINGLE LAIV | 1.286667e-05  (3.151677e-05) | 7.985683e-05  （0.0001447662） | 0.1611217 | 0.215 |
|  |  | NO VAC | 0.000000e+00  (0.000000e+00) | 0.000000e+00  (0.000000e+00) | NA | NA |
| M2 | H1N1 | PRIME BOOST | 9.392733e-05  (8.946395e-05) | 0.000000e+00  (0.000000e+00) | NA | 0.211 |
|  |  | SINGLE LAIV | 0.000000e+00  (0.000000e+00) | 0.0007105429  (0.00187992) | 0.000000 | 0.356 |
|  |  | NO VAC | 6.188571e-05  (1.637342e-04) | 0.000000e+00  (0.000000e+00) | NA | 0.356 |
|  | H3N2 | PRIME BOOST | 5.805209e-05  (1.530557e-04) | 0.000234388  (0.0007773771) | 0.2476752 | 0.487 |
|  |  | SINGLE LAIV | 5.169050e-05  (8.184563e-05) | 0.000000e+00  (0.000000e+00) | NA | 0.183 |
|  |  | NO VAC | 0.000000e+00  (0.000000e+00) | 0.000000e+00  (0.000000e+00) | NA | NA |
| NS1 | H1N1 | PRIME BOOST | 0.000000e+00  (0.000000e+00) | 3.912417e-04 (6.776504e-04) | 0.000000 | 0.423 |
|  |  | SINGLE LAIV | 7.927471e-05  (9.310807e-05) | 5.099057e-05  (8.721837e-05) | 1.554694 | 0.099 |
|  |  | NO VAC | 4.176514e-05  (5.918016e-05) | 4.273871e-05  (1.130760e-04) | 0.9772204 | 0.984 |
|  | H3N2 | PRIME BOOST | 1.106302e-04  (2.022070e-04) | 0.0006719935  (0.001021928) | 0.1646299 | 0.113 |
|  |  | SINGLE LAIV | 4.217833e-05  (6.994052e-05) | 0.000000e+00  (0.000000e+00) | NA | 0.200 |
|  |  | NO VAC | 3.687500e-05  (2.504201e-05) | 0.0000608675  (0.000121735) | 0.6058241 | 0.703 |
| NS2 | H1N1 | PRIME BOOST | 1.079553e-04 (1.869841e-04) | 0.000000e+00  (0.000000e+00) | NA | 0.423 |
|  |  | SINGLE LAIV | 5.814114e-05  (1.185236e-04) | 0.000219604  (0.0003008124) | 0.2647545 | 0.158 |
|  |  | NO VAC | 2.328571e-05  (3.978821e-05) | 0.001471748  (0.0026782477) | 0.01582181 | 0.205 |
|  | H3N2 | PRIME BOOST | 2.48981e-05  (5.679091e-05) | 0.0010487338  (0.002216130) | 0.02374111 | 0.180 |
|  |  | SINGLE LAIV | 0.000000e+00  (0.000000e+00) | 0.0004318657  (0.001057851) | 0.000000 | 0.363 |
|  |  | NO VAC | 1.40967e-04 (2.819340e-04) | 0.0001475828 (0.0002951655) | 0.9551723 | 0.979 |
