# Supplementary material for: Vaccination decreases the risk of influenza A virus reassortment but not genetic variation in pigs
Source: eLife. 2022 Sep 2;11:e78618. doi: 10.7554/eLife.78618 (PMC9439680; doi:10.7554/eLife.78618)
Supplement: Supplementary file 1. [file elife-78618-supp1.docx]

**Supplementary file 1. The comparison of humoral immune response in pigs whose bronchoalveolar lavage fluid (BALF) samples are available for direct sequencing or plaque purification between treatment groups.**

| **Category** | **Sample size^a^** | **Variable^b^** | **Group comparison** | **P value^c^** |
| --- | --- | --- | --- | --- |
| Direct sequencing (H1N1) | 19 | H1-specific hemagglutinin inhibition titer | PRIME BOOST – SINGLE LAIV | 0.0003 |
|  |  |  | PRIME BOOST – NO VAC | 0.0006 |
|  |  |  | SINGLE LAIV – NO VAC | 1.0000 |
| Direct sequencing (H3N2) | 21 | H3-specific hemagglutinin inhibition titer | PRIME BOOST – SINGLE LAIV | 0.0009 |
|  |  |  | PRIME BOOST – NO VAC | 0.0026 |
|  |  |  | SINGLE LAIV – NO VAC | 1.0000 |
| Plaque assay | 13 | H1-specific hemagglutinin inhibition titer | PRIME BOOST – SINGLE LAIV | 0.0214 |
|  |  |  | PRIME BOOST – NO VAC | 0.0427 |
|  |  |  | SINGLE LAIV – NO VAC | 1.0000 |
|  |  | H3-specific hemagglutinin inhibition titer | PRIME BOOST – SINGLE LAIV | 0.0066 |
|  |  |  | PRIME BOOST – NO VAC | 0.0132 |
|  |  |  | SINGLE LAIV – NO VAC | 1.0000 |

^a^ The columns of Sample size represent the number of BALF samples that successfully yielded the H1N1 genomes, H3N2 genomes, and IAV plaques, respectively, by direct sequencing or plaque assay.

^b^ The hemagglutinin inhibition assay was performed on blood samples from selected treatment pigs collected prior to contact with challenged pigs.

^c^ The means of hemagglutinin inhibition titers between treatment groups were compared by Kruskal-Wallis rank sum test. The Dunn’s test was utilized for the pairwise comparisons, the p values were adjusted using the Benjamini-Hochberg method.
