# Supplementary material for: Vaccination decreases the risk of influenza A virus reassortment but not genetic variation in pigs
Source: eLife. 2022 Sep 2;11:e78618. doi: 10.7554/eLife.78618 (PMC9439680; doi:10.7554/eLife.78618)
Supplement: Supplementary file 2. [file elife-78618-supp2.docx]

**Supplementary file 2. Number of broncho-alveolar lavage fluid (BALF) samples and number of plaques available for the study.**

| **Treatment** | **No. BALF samples** | **Pig/Sample ID** | **No. of plaques** | **TOTAL** |
| --- | --- | --- | --- | --- |
|  |  |  |  |  |
| **PRIME BOOST** | 5 | 4471 | 3 | **76** |
|  |  | 4551 | 20 |  |
|  |  | 5184 | 23 |  |
|  |  | 4469 | 1 |  |
|  |  | 4933 | 29 |  |
| **SINGLE LAIV** | 4 | 4479 | 17 | **52** |
|  |  | 4484 | 17 |  |
|  |  | 4945 | 2 |  |
|  |  | 5166 | 16 |  |
| **NO VAC** | 4 | 4490 | 24 | **74** |
|  |  | 5167 | 13 |  |
|  |  | 5174 | 23 |  |
|  |  | 5179 | 14 |  |
| **TOTAL** | **13** |  | **202** | |
