# Supplementary material for: Vaccination decreases the risk of influenza A virus reassortment but not genetic variation in pigs
Source: eLife. 2022 Sep 2;11:e78618. doi: 10.7554/eLife.78618 (PMC9439680; doi:10.7554/eLife.78618)
Supplement: Supplementary file 3. [file elife-78618-supp3.docx]

**Supplementary file 3. All identified functional single nucleotide variants (SNVs) in the H1N1 virus with annotations.**

| **Pig ID** | **Treatment** | **Coding region** | **Site** | **Reference** | **Variant** | **Frequency (%)** | **Amino acid change** | **Mutation type** | **Functional description** | **Functional type** |
| --- | --- | --- | --- | --- | --- | --- | --- | --- | --- | --- |
| 4473 | SINGLE LAIV | M1 | 598 | T | G | 88.5 | S200A | Non_Synonymous | M1 (C-terminal) vRNP binding region (Baudin, Petit, Weissenhorn, & Ruigrok, 2001) | Virus assembly, budding and release |
| 4486 | PRIME BOOST | M1 | 463 | G | A | 3.1 | A155T | Non_Synonymous | Associated with the virulence of H9 subtype virus in mice (Hui, Smee, Wong, & Nayak, 2006; Z. Ye, Liu, Offringa, McInnis, & Levandowski, 1999) | Determinant of pathogenicity, virulence and disease progression |
| 4486 | PRIME BOOST | M1 | 695 | A | G | 2 | D232G | Non_Synonymous | M1 (C-terminal) vRNP binding region (Baudin et al., 2001) | Virus assembly, budding and release |
| 4492 | SINGLE LAIV | M1 | 92 | T | C | 2 | V31A | Non_Synonymous | M1 (C-terminal) vRNP binding region (Baudin et al., 2001) | Virus assembly, budding and release |
| 4492 | SINGLE LAIV | M1 | 431 | T | C | 1.1 | F144S | Non_Synonymous | M1 (C-terminal) vRNP binding region (Baudin et al., 2001) | Virus assembly, budding and release |
| 4492 | SINGLE LAIV | M1 | 598 | T | G | 100 | S200A | Non_Synonymous | M1 (C-terminal) vRNP binding region (Baudin et al., 2001) | Virus assembly, budding and release |
| 4493 | NO VAC | M1 | 598 | T | G | 22.9 | S200A | Non_Synonymous | M1 (C-terminal) vRNP binding region (Baudin et al., 2001) | Virus assembly, budding and release |
| 4937 | SINGLE LAIV | M1 | 598 | T | G | 2.9 | S200A | Non_Synonymous | M1 (C-terminal) vRNP binding region (Baudin et al., 2001) | Virus assembly, budding and release |
| 4938 | NO VAC | M1 | 598 | T | G | 96.1 | S200A | Non_Synonymous | M1 (C-terminal) vRNP binding region (Baudin et al., 2001) | Virus assembly, budding and release |
| 4938 | NO VAC | M1 | 601 | G | A | 3.1 | E201K | Non_Synonymous | M1 (C-terminal) vRNP binding region (Baudin et al., 2001) | Virus assembly, budding and release |
| 5166 | SINGLE LAIV | M1 | 655 | A | G | 2.3 | I219V | Non_Synonymous | M1 (C-terminal) vRNP binding region (Baudin et al., 2001) | Virus assembly, budding and release |
| 5175 | PRIME BOOST | M1 | 587 | G | A | 7.7 | S196N | Non_Synonymous | M1 (C-terminal) vRNP binding region (Baudin et al., 2001) | Virus assembly, budding and release |
| 5175 | PRIME BOOST | M1 | 737 | T | C | 2.1 | V246A | Non_Synonymous | M1 (C-terminal) vRNP binding region (Baudin et al., 2001) | Virus assembly, budding and release |
| 5185 | PRIME BOOST | M1 | 383 | T | C | 1.2 | M128T | Non_Synonymous | M1 lipid/membrane binding region (Gregoriades & Frangione, 1981) | Virus assembly, budding and release |
| 5185 | PRIME BOOST | M1 | 732 | G | T | 1.2 | M244I | Non_Synonymous | M1 (C-terminal) vRNP binding region (Baudin et al., 2001) | Virus assembly, budding and release |
| 4479 | SINGLE LAIV | NA | 445 | G | A | 2.1 | V149I | Non_Synonymous | V149I is associated with support the NA active-site structure and decreased zanamivir sensitivity in a H5N1 virus (Naughtin et al., 2011; Yen et al., 2006) | Antiviral drug resistance |
| 4493 | NO VAC | NA | 1031 | A | G | 1.2 | N344S | Non_Synonymous | Binding site for zanamivir and oseltamivir, also associated with NA stability (Collins et al., 2008; Gupta et al., 2011; Van Der Vries et al., 2012) | Antiviral drug resistance |
| 4938 | NO VAC | NA | 169 | G | A | 1.5 | V57I | Non_Synonymous | Associated with the virulence of H5N1 in mice and chicken (Matsuoka et al., 2009; Zhou et al., 2009) | Determinant of pathogenicity, virulence and disease progression |
| 5175 | PRIME BOOST | NA | 626 | A | G | 1.5 | N209S | Non_Synonymous | Support the NA active-site structure (Yen et al., 2006) | Virus assembly, budding and release |
| 4479 | SINGLE LAIV | NP | 1490 | A | G | 1.2 | D497G | Non_Synonymous | Associated with PB2 interaction (Biswas, Boutz, & Nayak, 1998) | Viral genome/protein interaction |
| 4493 | NO VAC | NP | 183 | A | G | 5.7 | I61M | Non_Synonymous | Associated with RNA and PB2 interaction (Albo, Valencia, & Portela, 1995; Biswas et al., 1998; Elton, Medcalf, Bishop, Harrison, & Digard, 1999) | Viral genome/protein interaction |
| 4493 | NO VAC | NP | 845 | G | A | 1.8 | G282E | Non_Synonymous | Associated with PB2 interaction and NP oligomerization (Biswas et al., 1998; Elton, Medcalf, Bishop, & Digard, 1999) | Viral genome/protein interaction |
| 4938 | NO VAC | NP | 878 | G | A | 1.4 | R293K | Non_Synonymous | Associated with PB2 interaction and NP oligomerization (Biswas et al., 1998; Elton, Medcalf, Bishop, & Digard, 1999) | Viral genome/protein interaction |
| 4938 | NO VAC | NP | 992 | T | G | 1.1 | M331R | Non_Synonymous | Associated with PB2 interaction and NP oligomerization (Biswas et al., 1998; Elton, Medcalf, Bishop, & Digard, 1999) | Viral genome/protein interaction |
| 4938 | NO VAC | NP | 1278 | G | A | 1.7 | M426I | Non_Synonymous | Associated with PB2 interaction and NP oligomerization (Biswas et al., 1998; Elton, Medcalf, Bishop, & Digard, 1999; Q. Ye, Krug, & Tao, 2006) | Viral genome/protein interaction |
| 5174 | NO VAC | NP | 1066 | G | A | 15.6 | G356R | Non_Synonymous | Associated with PB2 interaction and NP oligomerization (Biswas et al., 1998; Elton, Medcalf, Bishop, & Digard, 1999) | Viral genome/protein interaction |
| 5175 | PRIME BOOST | NP | 425 | A | G | 1.4 | N142S | Non_Synonymous | Associated with RNA and PB2 interaction (Albo et al., 1995; Biswas et al., 1998; Elton, Medcalf, Bishop, Harrison, et al., 1999) | Viral genome/protein interaction |
| 5175 | PRIME BOOST | NP | 1112 | T | C | 2.2 | V371A | Non_Synonymous | Associated with PB2 interaction and NP oligomerization (Biswas et al., 1998; Elton, Medcalf, Bishop, & Digard, 1999) | Viral genome/protein interaction |
| 5185 | PRIME BOOST | NP | 886 | T | C | 44.4 | Y296H | Non_Synonymous | Associated with PB2 interaction and NP oligomerization (Biswas et al., 1998; Elton, Medcalf, Bishop, & Digard, 1999) | Viral genome/protein interaction |
| 5185 | PRIME BOOST | NP | 1256 | C | T | 91.7 | P419L | Non_Synonymous | Associated with PB2 interaction and NP oligomerization (Biswas et al., 1998; Elton, Medcalf, Bishop, & Digard, 1999; Q. Ye et al., 2006) | Viral genome/protein interaction |
| 4473 | SINGLE LAIV | NS1 | 374 | A | G | 1 | E125G | Non_Synonymous | A Gly at 125 confers high pathogenicity of H3N2 virus in mice and enhanced binding property to α-2,3 to α-2,6 sialic acid receptors. Also involved in multiple interactions with host proteins to meditate the host antiviral response (Bornholdt & Prasad, 2006; Das et al., 2008; Hale, Barclay, Randall, & Russell, 2008; Hale, Randall, Ortín, & Jackson, 2008; Narasaraju et al., 2009) | Determinant of pathogenicity, virulence and disease progression |
| 4473 | SINGLE LAIV | NS1 | 496 | C | A | 1.2 | L166I | Non_Synonymous | Associated with multiple host proteins interactions to meditate the host antiviral response (Bornholdt & Prasad, 2006; Das et al., 2008; Hale, Barclay, et al., 2008; Hale, Randall, et al., 2008) | Host-virus interaction machinery |
| 4479 | SINGLE LAIV | NS1 | 7 | T | C | 3.2 | S3P | Non_Synonymous | Associated with host proteins and dsRNA interactions to meditate the host antiviral response and IAV genome transportation (Cheng, Wong, & Yuan, 2009; Hale, Randall, et al., 2008; Yin et al., 2007) | Host-virus interaction machinery |
| 4479 | SINGLE LAIV | NS1 | 643 | C | T | 1.5 | P215S | Non_Synonymous | Associated with Crk/CrkL SH3 binding and CDK/ERK phosphorylation to meditate the host cell signaling (Hale, Barclay, et al., 2008; Hale et al., 2009; Heikkinen et al., 2008) | Host-virus interaction machinery |
| 4492 | SINGLE LAIV | NS1 | 619 | A | G | 1.5 | N207D | Non_Synonymous | Associated with Crk/CrkL SH3 binding and CDK/ERK phosphorylation to meditate the host cell signaling (Hale, Barclay, et al., 2008; Hale et al., 2009; Heikkinen et al., 2008) | Host-virus interaction machinery |
| 4493 | NO VAC | NS1 | 494 | C | A | 3.9 | S165Y | Non_Synonymous | Associated with multiple host protein interactions to meditate the host antiviral response (Bornholdt & Prasad, 2006; Das et al., 2008; Hale, Barclay, et al., 2008; Hale, Randall, et al., 2008) | Host-virus interaction machinery |
| 4934 | SINGLE LAIV | NS1 | 443 | G | A | 6.1 | R148K | Non_Synonymous | R148 is required for masking the nuclear export signal activity. Also involved in inhibiting the PI3K/Akt pathway and delay the host antiviral response (Bornholdt & Prasad, 2006; Das et al., 2008; Ehrhardt et al., 2007; Gallacher et al., 2009; Hale, Barclay, et al., 2008; Hale, Kerry, et al., 2010; Hale, Randall, et al., 2008; Y. Li, Yamakita, & Krug, 1998; Shin et al., 2007) | Viral genome transportation, transcription and replication |
| 4938 | NO VAC | NS1 | 100 | G | A | 1.3 | D34N | Non_Synonymous | Locate in RNA binding site and associated with host proteins and dsRNA interactions to meditate the host antiviral response and IAV genome transportation (Cheng et al., 2009; Hale, Randall, et al., 2008; Wang et al., 1999; Yin et al., 2007) | Host-virus interaction machinery |
| 5179 | NO VAC | NS1 | 196 | G | A | 1.2 | E66K | Non_Synonymous | Associated with multiple host proteins and dsRNA interactions to meditate the host antiviral response and IAV genome transportation (Cheng et al., 2009; Hale, Randall, et al., 2008; Yin et al., 2007) | Host-virus interaction machinery |
| 5179 | NO VAC | NS1 | 566 | G | A | 1.2 | G189D | Non_Synonymous | Associated with multiple host protein interactions to inhibit the processing the pre-mRNAs and host antiviral response (Bornholdt & Prasad, 2006; Das et al., 2008; Hale, Barclay, et al., 2008; Hale, Randall, et al., 2008; Hale, Steel, et al., 2010; Noah, Twu, & Krug, 2003; Twu, Kuo, Marklund, & Krug, 2007) | Host-virus interaction machinery |
| 4486 | PRIME BOOST | PA | 1034 | T | C | 1.7 | L345P | Non_Synonymous | PA (C-terminal) - PB1(N-terminal) binding region (He et al., 2008) | Viral genome/protein interaction |
| 4486 | PRIME BOOST | PA | 1255 | G | A | 4.3 | D419N | Non_Synonymous | PA (C-terminal) - PB1(N-terminal) binding region (He et al., 2008) | Viral genome/protein interaction |
| 4486 | PRIME BOOST | PA | 1780 | A | G | 1 | S594G | Non_Synonymous | PA (C-terminal) - PB1(N-terminal) binding region (He et al., 2008) | Viral genome/protein interaction |
| 4492 | SINGLE LAIV | PA | 906 | A | G | 1.3 | I302M | Non_Synonymous | PA (C-terminal) - PB1(N-terminal) binding region (He et al., 2008) | Viral genome/protein interaction |
| 4492 | SINGLE LAIV | PA | 2014 | C | T | 1.9 | L672F | Non_Synonymous | PA (C-terminal) - PB1(N-terminal) binding region (He et al., 2008) | Viral genome/protein interaction |
| 4492 | SINGLE LAIV | PA | 2137 | C | T | 3.5 | H713Y | Non_Synonymous | PA (C-terminal) - PB1(N-terminal) binding region (He et al., 2008) | Viral genome/protein interaction |
| 4493 | NO VAC | PA | 806 | G | A | 1.5 | R269K | Non_Synonymous | PA (C-terminal) - PB1(N-terminal) binding region (He et al., 2008) | Viral genome/protein interaction |
| 4493 | NO VAC | PA | 1949 | A | T | 7.5 | Y650F | Non_Synonymous | PA (C-terminal) - PB1(N-terminal) binding region (He et al., 2008) | Viral genome/protein interaction |
| 4495 | NO VAC | PA | 1051 | G | A | 32.7 | E351K | Non_Synonymous | PA (C-terminal) - PB1(N-terminal) binding region (He et al., 2008) | Viral genome/protein interaction |
| 4495 | NO VAC | PA | 1853 | C | T | 1.5 | T618I | Non_Synonymous | PA (C-terminal) - PB1(N-terminal) binding region (He et al., 2008) | Viral genome/protein interaction |
| 4499 | PRIME BOOST | PA | 1025 | T | C | 1 | L342P | Non_Synonymous | PA (C-terminal) - PB1(N-terminal) binding region (He et al., 2008) | Viral genome/protein interaction |
| 4938 | NO VAC | PA | 298 | G | A | 2.3 | V100I | Non_Synonymous | Inhibit the host protein synthesis and enhance the viral protein production (Desmet, Bussey, Stone, & Takimoto, 2013) | Host-virus interaction machinery |
| 5167 | NO VAC | PA | 1847 | C | T | 3 | S616L | Non_Synonymous | PA (C-terminal) - PB1(N-terminal) binding region (He et al., 2008) | Viral genome/protein interaction |
| 5174 | NO VAC | PA | 2121 | C | A | 1.1 | F707L | Non_Synonymous | PA (C-terminal) - PB1(N-terminal) binding region (He et al., 2008) | Viral genome/protein interaction |
| 5175 | PRIME BOOST | PA | 1388 | T | C | 1.1 | V463A | Non_Synonymous | PA (C-terminal) - PB1(N-terminal) binding region (He et al., 2008) | Viral genome/protein interaction |
| 5175 | PRIME BOOST | PA | 1834 | T | A | 6 | F612I | Non_Synonymous | PA (C-terminal) - PB1(N-terminal) binding region (He et al., 2008) | Viral genome/protein interaction |
| 4490 | NO VAC | PB1 | 2220 | C | A | 1.8 | F740L | Non_Synonymous | PB1 (C-terminal) - PB2 (N-terminal) binding region (Poole, Medcalf, Elton, & Digard, 2007; Sugiyama et al., 2009; Toyoda, Adyshev, Kobayashi, Iwata, & Ishihama, 1996) | Viral genome/protein interaction |
| 4492 | SINGLE LAIV | PB1 | 2087 | T | A | 1 | F696Y | Non_Synonymous | PB1 (C-terminal) - PB2 (N-terminal) binding region (Poole et al., 2007; Sugiyama et al., 2009; Toyoda et al., 1996) | Viral genome/protein interaction |
| 4493 | NO VAC | PB1 | 2168 | G | A | 30.8 | R723Q | Non_Synonymous | PB1 (C-terminal) - PB2 (N-terminal) binding region (Poole et al., 2007; Sugiyama et al., 2009; Toyoda et al., 1996) | Viral genome/protein interaction |
| 4495 | NO VAC | PB1 | 8 | T | C | 1.8 | V3A | Non_Synonymous | The introduction of V3A increased the virulence of a H5N1 virus in mice. Also locate in PB1 (N-terminal) – PA (C-terminal) interaction region (He et al., 2008; Salomon et al., 2006; Toyoda et al., 1996) | Determinant of pathogenicity, virulence and disease progression |
| 4495 | NO VAC | PB1 | 2110 | T | A | 2.2 | S704T | Non_Synonymous | PB1 (C-terminal) - PB2 (N-terminal) binding region (Poole et al., 2007; Sugiyama et al., 2009; Toyoda et al., 1996) | Viral genome/protein interaction |
| 4938 | NO VAC | PB1 | 2168 | G | A | 2.9 | R723Q | Non_Synonymous | PB1 (C-terminal) - PB2 (N-terminal) binding region (Poole et al., 2007; Sugiyama et al., 2009; Toyoda et al., 1996) | Viral genome/protein interaction |
| 5175 | PRIME BOOST | PB1 | 2098 | T | C | 2.7 | F700L | Non_Synonymous | PB1 (C-terminal) - PB2 (N-terminal) binding region (Poole et al., 2007; Sugiyama et al., 2009; Toyoda et al., 1996) | Viral genome/protein interaction |
| 4479 | SINGLE LAIV | PB2 | 1268 | G | A | 1.1 | R423K | Non_Synonymous | PB2 cap-binding region with m^7^GTP (Guilligay et al., 2008) | Viral genome transportation, transcription and replication |
| 4486 | PRIME BOOST | PB2 | 1267 | A | G | 1.4 | R423G | Non_Synonymous | PB2 cap-binding region with m^7^GTP (Guilligay et al., 2008) | Viral genome transportation, transcription and replication |
| 4486 | PRIME BOOST | PB2 | 1291 | A | G | 4.7 | M431V | Non_Synonymous | PB2 cap-binding region with m^7^GTP (Guilligay et al., 2008) | Viral genome transportation, transcription and replication |
| 4492 | SINGLE LAIV | PB2 | 1076 | G | A | 1.4 | G359E | Non_Synonymous | PB2 cap-binding region with m^7^GTP (Guilligay et al., 2008) | Viral genome transportation, transcription and replication |
| 4492 | SINGLE LAIV | PB2 | 1189 | G | A | 1.7 | A397T | Non_Synonymous | PB2 cap-binding region with m^7^GTP (Guilligay et al., 2008) | Viral genome transportation, transcription and replication |
| 4492 | SINGLE LAIV | PB2 | 2216 | G | T | 1.6 | R739L | Non_Synonymous | PB2 nuclear localization region (Mukaigawa & Nayak, 1991) | Viral genome transportation, transcription and replication |
| 4493 | NO VAC | PB2 | 1402 | G | A | 1.1 | A468T | Non_Synonymous | PB2 cap-binding region with m^7^GTP (Guilligay et al., 2008) | Viral genome transportation, transcription and replication |
| 5166 | SINGLE LAIV | PB2 | 2102 | A | G | 1.2 | D701G | Non_Synonymous | Essential mammal adapted site for avian influenza virus (Gao et al., 2009; Le, Sakai-Tagawa, Ozawa, Ito, & Kawaoka, 2009; Z. Li et al., 2005; Steel, Lowen, Mubareka, & Palese, 2009) | Cross-species transmission and adaption |
| 5167 | NO VAC | PB2 | 1396 | G | A | 47.1 | D466N | Non_Synonymous | PB2 nuclear localization region and cap-binding region with m^7^GTP (Guilligay et al., 2008; Mukaigawa & Nayak, 1991) | Viral genome transportation, transcription and replication |
| 5175 | PRIME BOOST | PB2 | 1138 | A | G | 2.1 | R380G | Non_Synonymous | PB2 cap-binding region with m^7^GTP (Guilligay et al., 2008) | Viral genome transportation, transcription and replication |
| 5175 | PRIME BOOST | PB2 | 2221 | T | C | 1.1 | S741P | Non_Synonymous | PB2 nuclear localization region (Mukaigawa & Nayak, 1991) | Viral genome transportation, transcription and replication |
| 5175 | PRIME BOOST | PB2 | 2257 | A | G | 1.2 | R753G | Non_Synonymous | PB2 nuclear localization region (Mukaigawa & Nayak, 1991) | Viral genome transportation, transcription and replication |

**Reference**

Albo, C., Valencia, A., & Portela, A. (1995). Identification of an RNA binding region within the N-terminal third of the influenza A virus nucleoprotein. *Journal of Virology*, *69*(6), 3799–3806.

Baudin, F., Petit, I., Weissenhorn, W., & Ruigrok, R. W. H. (2001). In vitro dissection of the membrane and RNP binding activities of influenza virus M1 protein. *Virology*, *281*(1), 102–108.

Biswas, S. K., Boutz, P. L., & Nayak, D. P. (1998). Influenza virus nucleoprotein interacts with influenza virus polymerase proteins. *Journal of Virology*, *72*(7), 5493–5501.

Bornholdt, Z. A., & Prasad, B. V. V. (2006). X-ray structure of influenza virus NS1 effector domain. *Nature Structural & Molecular Biology*, *13*(6), 559–560.

Cheng, A., Wong, S. M., & Yuan, Y. A. (2009). Structural basis for dsRNA recognition by NS1 protein of influenza A virus. *Cell Research*, *19*(2), 187–195.

Collins, P. J., Haire, L. F., Lin, Y. P., Liu, J., Russell, R. J., Walker, P. A., … Gamblin, S. J. (2008). Crystal structures of oseltamivir-resistant influenza virus neuraminidase mutants. *Nature*, *453*(7199), 1258–1261.

Das, K., Ma, L.-C., Xiao, R., Radvansky, B., Aramini, J., Zhao, L., … Arnold, E. (2008). Structural basis for suppression of a host antiviral response by influenza A virus. *Proceedings of the National Academy of Sciences*, *105*(35), 13093–13098.

Desmet, E. A., Bussey, K. A., Stone, R., & Takimoto, T. (2013). Identification of the N-terminal domain of the influenza virus PA responsible for the suppression of host protein synthesis. *Journal of Virology*, *87*(6), 3108–3118.

Ehrhardt, C., Wolff, T., Pleschka, S., Planz, O., Beermann, W., Bode, J. G., … Ludwig, S. (2007). Influenza A virus NS1 protein activates the PI3K/Akt pathway to mediate antiapoptotic signaling responses. *Journal of Virology*, *81*(7), 3058–3067.

Elton, D., Medcalf, E., Bishop, K., & Digard, P. (1999). Oligomerization of the influenza virus nucleoprotein: identification of positive and negative sequence elements. *Virology*, *260*(1), 190–200.

Elton, D., Medcalf, L., Bishop, K., Harrison, D., & Digard, P. (1999). Identification of amino acid residues of influenza virus nucleoprotein essential for RNA binding. *Journal of Virology*, *73*(9), 7357–7367.

Gallacher, M., Brown, S. G., Hale, B. G., Fearns, R., Olver, R. E., Randall, R. E., & Wilson, S. M. (2009). Cation currents in human airway epithelial cells induced by infection with influenza A virus. *The Journal of Physiology*, *587*(13), 3159–3173.

Gao, Y., Zhang, Y., Shinya, K., Deng, G., Jiang, Y., Li, Z., … Shi, J. (2009). Identification of amino acids in HA and PB2 critical for the transmission of H5N1 avian influenza viruses in a mammalian host. *PLoS Pathog*, *5*(12), e1000709.

Gregoriades, A., & Frangione, B. (1981). Insertion of influenza M protein into the viral lipid bilayer and localization of site of insertion. *Journal of Virology*, *40*(1), 323–328.

Guilligay, D., Tarendeau, F., Resa-Infante, P., Coloma, R., Crepin, T., Sehr, P., … Hart, D. J. (2008). The structural basis for cap binding by influenza virus polymerase subunit PB2. *Nature Structural & Molecular Biology*, *15*(5), 500.

Gupta, S. K., Gupta, S. K., Smita, S., Srivastava, M., Lai, X., Schmitz, U., … Vera, J. (2011). Computational analysis and modeling the effectiveness of ‘Zanamivir’targeting neuraminidase protein in pandemic H1N1 strains. *Infection, Genetics and Evolution*, *11*(5), 1072–1082.

Hale, B. G., Barclay, W. S., Randall, R. E., & Russell, R. J. (2008). Structure of an avian influenza A virus NS1 protein effector domain. *Virology*, *378*(1), 1–5.

Hale, B. G., Kerry, P. S., Jackson, D., Precious, B. L., Gray, A., Killip, M. J., … Russell, R. J. (2010). Structural insights into phosphoinositide 3-kinase activation by the influenza A virus NS1 protein. *Proceedings of the National Academy of Sciences*, *107*(5), 1954–1959.

Hale, B. G., Knebel, A., Botting, C. H., Galloway, C. S., Precious, B. L., Jackson, D., … Randall, R. E. (2009). CDK/ERK-mediated phosphorylation of the human influenza A virus NS1 protein at threonine-215. *Virology*, *383*(1), 6–11.

Hale, B. G., Randall, R. E., Ortín, J., & Jackson, D. (2008). The multifunctional NS1 protein of influenza A viruses. *Journal of General Virology*, *89*(10), 2359–2376.

Hale, B. G., Steel, J., Medina, R. A., Manicassamy, B., Ye, J., Hickman, D., … Perez, D. R. (2010). Inefficient control of host gene expression by the 2009 pandemic H1N1 influenza A virus NS1 protein. *Journal of Virology*, *84*(14), 6909–6922.

He, X., Zhou, J., Bartlam, M., Zhang, R., Ma, J., Lou, Z., … Zeng, Z. (2008). Crystal structure of the polymerase PA C–PB1 N complex from an avian influenza H5N1 virus. *Nature*, *454*(7208), 1123–1126.

Heikkinen, L. S., Kazlauskas, A., Melén, K., Wagner, R., Ziegler, T., Julkunen, I., & Saksela, K. (2008). Avian and 1918 Spanish influenza a virus NS1 proteins bind to Crk/CrkL Src homology 3 domains to activate host cell signaling. *Journal of Biological Chemistry*, *283*(9), 5719–5727.

Hui, E. K.-W., Smee, D. F., Wong, M.-H., & Nayak, D. P. (2006). Mutations in influenza virus M1 CCHH, the putative zinc finger motif, cause attenuation in mice and protect mice against lethal influenza virus infection. *Journal of Virology*, *80*(12), 5697–5707.

Le, Q. M., Sakai-Tagawa, Y., Ozawa, M., Ito, M., & Kawaoka, Y. (2009). Selection of H5N1 influenza virus PB2 during replication in humans. *Journal of Virology*, *83*(10), 5278–5281.

Li, Y., Yamakita, Y., & Krug, R. M. (1998). Regulation of a nuclear export signal by an adjacent inhibitory sequence: the effector domain of the influenza virus NS1 protein. *Proceedings of the National Academy of Sciences*, *95*(9), 4864–4869.

Li, Z., Chen, H., Jiao, P., Deng, G., Tian, G., Li, Y., … Yu, K. (2005). Molecular basis of replication of duck H5N1 influenza viruses in a mammalian mouse model. *Journal of Virology*, *79*(18), 12058–12064.

Matsuoka, Y., Swayne, D. E., Thomas, C., Rameix-Welti, M.-A., Naffakh, N., Warnes, C., … Subbarao, K. (2009). Neuraminidase stalk length and additional glycosylation of the hemagglutinin influence the virulence of influenza H5N1 viruses for mice. *Journal of Virology*, *83*(9), 4704–4708.

Mukaigawa, J., & Nayak, D. P. (1991). Two signals mediate nuclear localization of influenza virus (A/WSN/33) polymerase basic protein 2. *Journal of Virology*, *65*(1), 245–253.

Narasaraju, T., Sim, M. K., Ng, H. H., Phoon, M. C., Shanker, N., Lal, S. K., & Chow, V. T. K. (2009). Adaptation of human influenza H3N2 virus in a mouse pneumonitis model: insights into viral virulence, tissue tropism and host pathogenesis. *Microbes and Infection*, *11*(1), 2–11.

Naughtin, M., Dyason, J. C., Mardy, S., Sorn, S., Von Itzstein, M., & Buchy, P. (2011). Neuraminidase inhibitor sensitivity and receptor-binding specificity of Cambodian clade 1 highly pathogenic H5N1 influenza virus. *Antimicrobial Agents and Chemotherapy*, *55*(5), 2004–2010.

Noah, D. L., Twu, K. Y., & Krug, R. M. (2003). Cellular antiviral responses against influenza A virus are countered at the posttranscriptional level by the viral NS1A protein via its binding to a cellular protein required for the 3′ end processing of cellular pre-mRNAS. *Virology*, *307*(2), 386–395.

Poole, E. L., Medcalf, L., Elton, D., & Digard, P. (2007). Evidence that the C-terminal PB2-binding region of the influenza A virus PB1 protein is a discrete α-helical domain. *FEBS Letters*, *581*(27), 5300–5306.

Salomon, R., Franks, J., Govorkova, E. A., Ilyushina, N. A., Yen, H.-L., Hulse-Post, D. J., … Webby, R. J. (2006). The polymerase complex genes contribute to the high virulence of the human H5N1 influenza virus isolate A/Vietnam/1203/04. *Journal of Experimental Medicine*, *203*(3), 689–697.

Shin, Y.-K., Li, Y., Liu, Q., Anderson, D. H., Babiuk, L. A., & Zhou, Y. (2007). SH3 binding motif 1 in influenza A virus NS1 protein is essential for PI3K/Akt signaling pathway activation. *Journal of Virology*, *81*(23), 12730–12739.

Steel, J., Lowen, A. C., Mubareka, S., & Palese, P. (2009). Transmission of influenza virus in a mammalian host is increased by PB2 amino acids 627K or 627E/701N. *PLoS Pathogens*, *5*(1).

Sugiyama, K., Obayashi, E., Kawaguchi, A., Suzuki, Y., Tame, J. R. H., Nagata, K., & Park, S. (2009). Structural insight into the essential PB1–PB2 subunit contact of the influenza virus RNA polymerase. *The EMBO Journal*, *28*(12), 1803–1811.

Toyoda, T., Adyshev, D. M., Kobayashi, M., Iwata, A., & Ishihama, A. (1996). Molecular assembly of the influenza virus RNA polymerase: determination of the subunit-subunit contact sites. *Journal of General Virology*, *77*(9), 2149–2157.

Twu, K. Y., Kuo, R.-L., Marklund, J., & Krug, R. M. (2007). The H5N1 influenza virus NS genes selected after 1998 enhance virus replication in mammalian cells. *Journal of Virology*, *81*(15), 8112–8121.

Van Der Vries, E., Collins, P. J., Vachieri, S. G., Xiong, X., Liu, J., Walker, P. A., … Osterhaus, A. D. M. E. (2012). H1N1 2009 pandemic influenza virus: resistance of the I223R neuraminidase mutant explained by kinetic and structural analysis. *PLoS Pathog*, *8*(9), e1002914.

Wang, W., Riedel, K., Lynch, P., Chien, C. Y., Montelione, G. T., & Krug, R. M. (1999). RNA binding by the novel helical domain of the influenza virus NS1 protein requires its dimer structure and a small number of specific basic amino acids. *Rna*, *5*(2), 195–205.

Ye, Q., Krug, R. M., & Tao, Y. J. (2006). The mechanism by which influenza A virus nucleoprotein forms oligomers and binds RNA. *Nature*, *444*(7122), 1078–1082.

Ye, Z., Liu, T., Offringa, D. P., McInnis, J., & Levandowski, R. A. (1999). Association of influenza virus matrix protein with ribonucleoproteins. *Journal of Virology*, *73*(9), 7467–7473.

Yen, H.-L., Hoffmann, E., Taylor, G., Scholtissek, C., Monto, A. S., Webster, R. G., & Govorkova, E. A. (2006). Importance of neuraminidase active-site residues to the neuraminidase inhibitor resistance of influenza viruses. *Journal of Virology*, *80*(17), 8787–8795.

Yin, C., Khan, J. A., Swapna, G. V. T., Ertekin, A., Krug, R. M., Tong, L., & Montelione, G. T. (2007). Conserved surface features form the double-stranded RNA binding site of non-structural protein 1 (NS1) from influenza A and B viruses. *Journal of Biological Chemistry*, *282*(28), 20584–20592.

Zhou, H., Yu, Z., Hu, Y., Tu, J., Zou, W., Peng, Y., … Yu, Z. (2009). The special neuraminidase stalk-motif responsible for increased virulence and pathogenesis of H5N1 influenza A virus. *PloS One*, *4*(7), e6277.
