# Supplementary material for: Vaccination decreases the risk of influenza A virus reassortment but not genetic variation in pigs
Source: eLife. 2022 Sep 2;11:e78618. doi: 10.7554/eLife.78618 (PMC9439680; doi:10.7554/eLife.78618)
Supplement: Supplementary file 4. [file elife-78618-supp4.docx]

**Supplementary file 4. All identified functional single nucleotide variants (SNVs) in the H3N2 virus with annotations.**

| **Pig ID** | **Treatment** | **Coding region** | **Site** | **Reference** | **Variant** | **Frequency (%)** | **Amino acid change** | **Mutation type** | **Functional description** | **Functional type** |
| --- | --- | --- | --- | --- | --- | --- | --- | --- | --- | --- |
| 4467 | PRIME BOOST | M1 | 637 | G | A | 2.8 | V213I | Non_Synonymous | M1 (C-terminal) vRNP binding region (Baudin, Petit, Weissenhorn, & Ruigrok, 2001) | Virus assembly, budding and release |
| 4486 | PRIME BOOST | M1 | 389 | T | C | 2.2 | L130P | Non_Synonymous | M1 lipid/membrane binding region (Baudin et al., 2001; Gregoriades & Frangione, 1981) | Virus assembly, budding and release |
| 4499 | PRIME BOOST | M1 | 406 | G | A | 1.3 | G136R | Non_Synonymous | M1 lipid/membrane binding region (Baudin et al., 2001; Z. Ye, Liu, Offringa, McInnis, & Levandowski, 1999) | Virus assembly, budding and release |
| 4499 | PRIME BOOST | M1 | 551 | C | A | 1.3 | T184N | Non_Synonymous | M1 (C-terminal) vRNP binding region (Baudin et al., 2001) | Virus assembly, budding and release |
| 4552 | PRIME BOOST | M1 | 43 | A | G | 3.4 | I15V | Non_Synonymous | M1 (N-terminal) vRNP binding region (Baudin et al., 2001; Z. Ye et al., 1999) | Virus assembly, budding and release |
| 4552 | PRIME BOOST | M1 | 128 | T | C | 31 | M43T | Non_Synonymous | M1 (N-terminal) vRNP binding region (Baudin et al., 2001; Z. Ye et al., 1999) | Virus assembly, budding and release |
| 4552 | PRIME BOOST | M1 | 529 | A | G | 1.5 | N177D | Non_Synonymous | M1 (C-terminal) vRNP binding region (Baudin et al., 2001) | Virus assembly, budding and release |
| 4931 | PRIME BOOST | M2 | 836 | T | C | 1.1 | C50R | Non_Synonymous | C50 is a palmitoylation site which associated with virus virulence in mouse and M2 cholesterol binding (Grantham et al., 2009; Schroeder, Heider, Möncke-Buchner, & Lin, 2005) | Determinant of pathogenicity, virulence and disease progression |
| 4934 | SINGLE LAIV | M1 | 476 | A | T | 1.1 | H159L | Non_Synonymous | Associated with the virulence of H9 subtype virus in mice (Baudin et al., 2001; Hui, Smee, Wong, & Nayak, 2006; Z. Ye et al., 1999) | Determinant of pathogenicity, virulence and disease progression |
| 4934 | SINGLE LAIV | M1 | 590 | A | G | 1.1 | E197G | Non_Synonymous | M1 (C-terminal) vRNP binding region (Baudin et al., 2001) | Virus assembly, budding and release |
| 4467 | PRIME BOOST | NP | 127 | A | G | 3.6 | M43V | Non_Synonymous | Associated with RNA and PB2 interaction (Albo, Valencia, & Portela, 1995; Biswas, Boutz, & Nayak, 1998; Elton, Medcalf, Bishop, Harrison, & Digard, 1999) | Viral genome/protein interaction |
| 4467 | PRIME BOOST | NP | 448 | A | G | 1.8 | R150G | Non_Synonymous | Associated with RNA and PB2 interaction (Albo et al., 1995; Biswas et al., 1998; Elton, Medcalf, Bishop, Harrison, et al., 1999) | Viral genome/protein interaction |
| 4467 | PRIME BOOST | NP | 878 | G | A | 1.1 | R293K | Non_Synonymous | Associated with PB2 interaction and NP oligomerization (Biswas et al., 1998; Elton, Medcalf, Bishop, & Digard, 1999) | Viral genome/protein interaction |
| 4467 | PRIME BOOST | NP | 1244 | A | G | 1.4 | Q415R | Non_Synonymous | Associated with NP oligomerization (Biswas et al., 1998; Elton, Medcalf, Bishop, & Digard, 1999; Q. Ye, Krug, & Tao, 2006) | Viral genome/protein interaction |
| 4469 | PRIME BOOST | NP | 115 | T | C | 2.6 | F39L | Non_Synonymous | Associated with RNA and PB2 interaction (Albo et al., 1995; Biswas et al., 1998; Elton, Medcalf, Bishop, Harrison, et al., 1999) | Viral genome/protein interaction |
| 4469 | PRIME BOOST | NP | 1341 | G | C | 1 | M447I | Non_Synonymous | Associated with PB2 interaction and NP oligomerization (Biswas et al., 1998; Elton, Medcalf, Bishop, & Digard, 1999) | Viral genome/protein interaction |
| 4479 | SINGLE LAIV | NP | 1288 | A | G | 1.2 | S430G | Non_Synonymous | Associated with PB2 interaction and NP oligomerization (Biswas et al., 1998; Elton, Medcalf, Bishop, & Digard, 1999) | Viral genome/protein interaction |
| 4486 | PRIME BOOST | NP | 189 | A | G | 1.8 | I63M | Non_Synonymous | Associated with RNA and PB2 interaction (Albo et al., 1995; Biswas et al., 1998; Elton, Medcalf, Bishop, Harrison, et al., 1999) | Viral genome/protein interaction |
| 4486 | PRIME BOOST | NP | 401 | C | G | 39.2 | T134S | Non_Synonymous | Associated with RNA and PB2 interaction (Albo et al., 1995; Biswas et al., 1998; Elton, Medcalf, Bishop, Harrison, et al., 1999) | Viral genome/protein interaction |
| 4486 | PRIME BOOST | NP | 406 | A | C | 1.4 | I136L | Non_Synonymous | Associated with RNA and PB2 interaction (Albo et al., 1995; Biswas et al., 1998; Elton, Medcalf, Bishop, Harrison, et al., 1999) | Viral genome/protein interaction |
| 4486 | PRIME BOOST | NP | 469 | A | G | 1.2 | T157A | Non_Synonymous | Associated with RNA and PB2 interaction (Albo et al., 1995; Biswas et al., 1998; Elton, Medcalf, Bishop, Harrison, et al., 1999) | Viral genome/protein interaction |
| 4490 | NO VAC | NP | 293 | G | A | 2.4 | R98K | Non_Synonymous | Associated with RNA and PB2 interaction (Albo et al., 1995; Biswas et al., 1998; Elton, Medcalf, Bishop, Harrison, et al., 1999) | Viral genome/protein interaction |
| 4499 | PRIME BOOST | NP | 644 | C | A | 1.2 | T215K | Non_Synonymous | Associated with PB2 interaction and NP nuclear localization (Elton, Medcalf, Bishop, & Digard, 1999; Weber, Kochs, Gruber, & Haller, 1998) | Host-virus interaction machinery |
| 4552 | PRIME BOOST | NP | 203 | T | C | 1.3 | L68P | Non_Synonymous | Associated with RNA and PB2 interaction (Albo et al., 1995; Biswas et al., 1998; Elton, Medcalf, Bishop, Harrison, et al., 1999) | Viral genome/protein interaction |
| 4552 | PRIME BOOST | NP | 464 | T | C | 3.1 | V155A | Non_Synonymous | Associated with RNA and PB2 interaction (Albo et al., 1995; Biswas et al., 1998; Elton, Medcalf, Bishop, Harrison, et al., 1999) | Viral genome/protein interaction |
| 4552 | PRIME BOOST | NP | 731 | A | G | 1.6 | E244G | Non_Synonymous | Associated with NP oligomerization (Elton, Medcalf, Bishop, & Digard, 1999) | Viral genome/protein interaction |
| 4552 | PRIME BOOST | NP | 839 | T | C | 1.4 | V280A | Non_Synonymous | Associated with PB2 interaction and NP oligomerization (Biswas et al., 1998; Elton, Medcalf, Bishop, & Digard, 1999) | Viral genome/protein interaction |
| 4552 | PRIME BOOST | NP | 1373 | T | C | 1.3 | F458S | Non_Synonymous | Associated with PB2 interaction and NP oligomerization (Biswas et al., 1998; Elton, Medcalf, Bishop, & Digard, 1999) | Viral genome/protein interaction |
| 4931 | PRIME BOOST | NP | 362 | G | A | 1.4 | R121H | Non_Synonymous | Associated with RNA and PB2 interaction (Albo et al., 1995; Biswas et al., 1998; Elton, Medcalf, Bishop, Harrison, et al., 1999) | Viral genome/protein interaction |
| 4931 | PRIME BOOST | NP | 439 | A | G | 3 | T147A | Non_Synonymous | Associated with RNA and PB2 interaction (Albo et al., 1995; Biswas et al., 1998; Elton, Medcalf, Bishop, Harrison, et al., 1999) | Viral genome/protein interaction |
| 4945 | SINGLE LAIV | NP | 1117 | G | A | 2.2 | A373T | Non_Synonymous | Associated with PB2 interaction and NP oligomerization (Biswas et al., 1998; Elton, Medcalf, Bishop, & Digard, 1999) | Viral genome/protein interaction |
| 5167 | NO VAC | NP | 181 | A | C | 2.8 | I61L | Non_Synonymous | Associated with RNA and PB2 interaction (Albo et al., 1995; Biswas et al., 1998; Elton, Medcalf, Bishop, Harrison, et al., 1999) | Viral genome/protein interaction |
| 5184 | PRIME BOOST | NP | 388 | A | G | 3.6 | T130A | Non_Synonymous | Associated with RNA and PB2 interaction (Albo et al., 1995; Biswas et al., 1998; Elton, Medcalf, Bishop, Harrison, et al., 1999) | Viral genome/protein interaction |
| 4467 | PRIME BOOST | NS1 | 637 | T | C | 15.9 | S213P | Non_Synonymous | Associated with Crk/CrkL SH3 binding and CDK/ERK phosphorylation to meditate the host cell signaling (Hale, Barclay, Randall, & Russell, 2008; Hale et al., 2009) | Host-virus interaction machinery |
| 4469 | PRIME BOOST | NS1 | 309 | C | A | 3.7 | F103L | Non_Synonymous | A F103L substitution in a H5N1 virus exhibit increased virulence and expand tissue-tropism in mice. Also associated with multiple host protein interactions to inhibit the processing the pre-mRNAs and host antiviral response (Aragón et al., 2000; Bornholdt & Prasad, 2006; Das et al., 2008; Hale, Barclay, et al., 2008; Hale, Randall, Ortín, & Jackson, 2008; Hale, Steel, et al., 2010; Kochs, García-Sastre, & Martínez-Sobrido, 2007; Noah, Twu, & Krug, 2003; Twu, Kuo, Marklund, & Krug, 2007) | Determinant of pathogenicity, virulence and disease progression |
| 4481 | SINGLE LAIV | NS1 | 251 | T | C | 4.4 | V84A | Non_Synonymous | The deletion of the region confers increased virulence of virus in mice and chicken. Also involved in host protein interaction for viral protein production (Aragón et al., 2000; Bornholdt & Prasad, 2008; Long, Peng, Liu, Wu, & Liu, 2008) | Determinant of pathogenicity, virulence and disease progression |
| 4490 | NO VAC | NS1 | 572 | C | T | 1.4 | T191M | Non_Synonymous | Associated with multiple host proteins interactions to meditate the host antiviral response (Bornholdt & Prasad, 2006; Das et al., 2008; Hale, Barclay, et al., 2008; Hale, Randall, et al., 2008) | Host-virus interaction machinery |
| 4490 | NO VAC | NS2 | 736 | A | C | 7.3 | K88N | Non_Synonymous | NS2 nuclear localization region (Shimizu, Takizawa, Watanabe, Nagata, & Kobayashi, 2011) | Viral genome transportation, transcription and replication |
| 4552 | PRIME BOOST | NS1 | 41 | T | C | 8.7 | F14S | Non_Synonymous | Associated with host proteins and dsRNA interactions to meditate the host antiviral response and IAV genome transportation (Cheng, Wong, & Yuan, 2009; Hale, Randall, et al., 2008; Yin et al., 2007) | Host-virus interaction machinery |
| 4552 | PRIME BOOST | NS1 | 256 | A | G | 1.3 | T86A | Non_Synonymous | The deletion of the region confers increased virulence of virus (H1N1 and H5N1) in mice and chicken. Also involved in host protein interaction for viral protein production (Aragón et al., 2000; Bornholdt & Prasad, 2008; Long et al., 2008) | Host-virus interaction machinery |
| 4552 | PRIME BOOST | NS1 | 409 | A | G | 1.6 | I137V | Non_Synonymous | Involved in inhibiting the PI3K/Akt pathway and delay the host antiviral response (Ehrhardt et al., 2007; Gallacher et al., 2009; Hale, Jackson, Chen, Lamb, & Randall, 2006; Hale, Kerry, et al., 2010; Li, Yamakita, & Krug, 1998; Shin et al., 2007) | Host-virus interaction machinery |
| 4934 | SINGLE LAIV | NS1 | 455 | A | G | 2.2 | E152G | Non_Synonymous | E152 is required for masking the nuclear export signal activity. Also involved in inhibiting the PI3K/Akt pathway and delay the host antiviral response (Bornholdt & Prasad, 2006; Das et al., 2008; Ehrhardt et al., 2007; Hale, Barclay, et al., 2008; Hale, Randall, et al., 2008) | Viral genome transportation, transcription and replication |
| 5167 | NO VAC | NS1 | 239 | C | T | 1.3 | T80I | Non_Synonymous | The deletion of the region confers increased virulence of virus (H1N1 and H5N1) in mice and chicken (Bornholdt & Prasad, 2008; Long et al., 2008; Seo, Hoffmann, & Webster, 2002) | Determinant of pathogenicity, virulence and disease progression |
| 5174 | NO VAC | NS1 | 157 | G | T | 1.1 | D53Y | Non_Synonymous | Associated with host proteins and dsRNA interactions to meditate the host antiviral response and IAV genome transportation (Cheng et al., 2009; Hale, Randall, et al., 2008; Yin et al., 2007) | Host-virus interaction machinery |
| 4499 | PRIME BOOST | HA | 14 | T | C | 2.4 | I5T | Non_Synonymous | Hemagglutinin signal peptide region (Air, 1979) | Viral genome transportation, transcription and replication |
| 4479 | SINGLE LAIV | PA | 1783 | A | G | 1.2 | M595V | Non_Synonymous | PA (C-terminal) - PB1(N-terminal) binding region (He et al., 2008) | Viral genome/protein interaction |
| 4486 | PRIME BOOST | PA | 193 | T | C | 17.3 | S65P | Non_Synonymous | Involved in suppressing the host protein synthesis and enhance the viral protein production (Desmet, Bussey, Stone, & Takimoto, 2013) | Host-virus interaction machinery |
| 4490 | NO VAC | PA | 1987 | A | G | 1.7 | R663G | Non_Synonymous | PA (C-terminal) - PB1(N-terminal) binding region (He et al., 2008) | Viral genome/protein interaction |
| 4552 | PRIME BOOST | PA | 803 | T | C | 1.3 | L268P | Non_Synonymous | PA (C-terminal) - PB1(N-terminal) binding region (He et al., 2008) | Viral genome/protein interaction |
| 4552 | PRIME BOOST | PA | 1268 | T | C | 2.3 | M423T | Non_Synonymous | PA (C-terminal) - PB1(N-terminal) binding region (He et al., 2008) | Viral genome/protein interaction |
| 4552 | PRIME BOOST | PA | 1363 | G | A | 5.2 | A455T | Non_Synonymous | PA (C-terminal) - PB1(N-terminal) binding region (He et al., 2008) | Viral genome/protein interaction |
| 4552 | PRIME BOOST | PA | 1576 | T | C | 3.7 | S526P | Non_Synonymous | PA (C-terminal) - PB1(N-terminal) binding region (He et al., 2008) | Viral genome/protein interaction |
| 4552 | PRIME BOOST | PA | 1805 | T | C | 1.2 | V602A | Non_Synonymous | PA (C-terminal) - PB1(N-terminal) binding region (He et al., 2008) | Viral genome/protein interaction |
| 4931 | PRIME BOOST | PA | 881 | A | G | 1.1 | D294G | Non_Synonymous | PA (C-terminal) - PB1(N-terminal) binding region (He et al., 2008) | Viral genome/protein interaction |
| 4931 | PRIME BOOST | PA | 1030 | G | A | 8.6 | E344K | Non_Synonymous | PA (C-terminal) - PB1(N-terminal) binding region (He et al., 2008) | Viral genome/protein interaction |
| 4934 | SINGLE LAIV | PA | 1183 | A | G | 9.2 | S395G | Non_Synonymous | PA (C-terminal) - PB1(N-terminal) binding region (He et al., 2008) | Viral genome/protein interaction |
| 4934 | SINGLE LAIV | PA | 1963 | C | T | 1.7 | L655F | Non_Synonymous | PA (C-terminal) - PB1(N-terminal) binding region (He et al., 2008) | Viral genome/protein interaction |
| 4934 | SINGLE LAIV | PA | 2129 | T | C | 2 | F710S | Non_Synonymous | PA (C-terminal) - PB1(N-terminal) binding region (He et al., 2008) | Viral genome/protein interaction |
| 5166 | SINGLE LAIV | PA | 1574 | T | C | 1 | F525S | Non_Synonymous | PA (C-terminal) - PB1(N-terminal) binding region (He et al., 2008) | Viral genome/protein interaction |
| 5167 | NO VAC | PA | 2014 | C | A | 3.8 | L672I | Non_Synonymous | PA (C-terminal) - PB1(N-terminal) binding region (He et al., 2008) | Viral genome/protein interaction |
| 5179 | NO VAC | PA | 1678 | C | T | 1.4 | P560S | Non_Synonymous | PA (C-terminal) - PB1(N-terminal) binding region (He et al., 2008) | Viral genome/protein interaction |
| 5185 | PRIME BOOST | PA | 1662 | A | G | 1.1 | I554M | Non_Synonymous | PA (C-terminal) - PB1(N-terminal) binding region (He et al., 2008) | Viral genome/protein interaction |
| 5185 | PRIME BOOST | PA | 2120 | T | C | 9.2 | F707S | Non_Synonymous | PA (C-terminal) - PB1(N-terminal) binding region (He et al., 2008) | Viral genome/protein interaction |
| 4481 | SINGLE LAIV | PB1 | 2110 | T | C | 1.1 | S704P | Non_Synonymous | PB1 (C-terminal) - PB2 (N-terminal) binding region (Poole, Medcalf, Elton, & Digard, 2007; Sugiyama et al., 2009; Toyoda, Adyshev, Kobayashi, Iwata, & Ishihama, 1996) | Viral genome/protein interaction |
| 4486 | PRIME BOOST | PB1 | 2162 | G | A | 1.6 | R721K | Non_Synonymous | PB1 (C-terminal) - PB2 (N-terminal) binding region (Poole et al., 2007; Sugiyama et al., 2009; Toyoda et al., 1996) | Viral genome/protein interaction |
| 4486 | PRIME BOOST | PB1 | 2224 | G | A | 1.2 | E742K | Non_Synonymous | PB1 (C-terminal) - PB2 (N-terminal) binding region (Poole et al., 2007; Sugiyama et al., 2009; Toyoda et al., 1996) | Viral genome/protein interaction |
| 4551 | PRIME BOOST | PB1 | 632 | G | A | 99.9 | R211K | Non_Synonymous | PB1 nuclear localization region | Viral genome transportation, transcription and replication |
| 4552 | PRIME BOOST | PB1 | 613 | A | G | 9 | I205V | Non_Synonymous | PB1 nuclear localization region | Viral genome transportation, transcription and replication |
| 4552 | PRIME BOOST | PB1 | 2208 | G | T | 1.4 | K736N | Non_Synonymous | PB1 (C-terminal) - PB2 (N-terminal) binding region (Poole et al., 2007; Sugiyama et al., 2009; Toyoda et al., 1996) | Viral genome/protein interaction |
| 4931 | PRIME BOOST | PB1 | 571 | A | G | 1.2 | M191V | Non_Synonymous | PB1 nuclear localization region | Viral genome transportation, transcription and replication |
| 4934 | SINGLE LAIV | PB1 | 2270 | A | G | 1.4 | K757R | Non_Synonymous | PB1 (C-terminal) - PB2 (N-terminal) binding region (Poole et al., 2007; Sugiyama et al., 2009; Toyoda et al., 1996) | Viral genome/protein interaction |
| 5167 | NO VAC | PB1 | 2095 | T | C | 1.1 | F699L | Non_Synonymous | PB1 (C-terminal) - PB2 (N-terminal) binding region (Poole et al., 2007; Sugiyama et al., 2009; Toyoda et al., 1996) | Viral genome/protein interaction |
| 5184 | PRIME BOOST | PB1 | 713 | G | A | 2.1 | R238K | Non_Synonymous | Promoter binding site for influenza polymerse (Jung & Brownlee, 2006) | Viral genome transportation, transcription and replication |
| 5185 | PRIME BOOST | PB1 | 758 | A | G | 1.1 | Y253C | Non_Synonymous | PB1 nuclear localization region | Viral genome transportation, transcription and replication |
| 4467 | PRIME BOOST | PB2 | 1030 | G | A | 1.2 | V344M | Non_Synonymous | PB2 cap-binding region with m^7^GTP (Guilligay et al., 2008) | Viral genome transportation, transcription and replication |
| 4467 | PRIME BOOST | PB2 | 1097 | T | C | 1.9 | V366A | Non_Synonymous | PB2 cap-binding region with m^7^GTP (Guilligay et al., 2008) | Viral genome transportation, transcription and replication |
| 4479 | SINGLE LAIV | PB2 | 2216 | G | T | 1.2 | R739L | Non_Synonymous | PB2 nuclear localization region (Mukaigawa & Nayak, 1991) | Viral genome transportation, transcription and replication |
| 4481 | SINGLE LAIV | PB2 | 985 | A | G | 1.4 | T329A | Non_Synonymous | PB2 cap-binding region with m^7^GTP (Guilligay et al., 2008) | Viral genome transportation, transcription and replication |
| 4481 | SINGLE LAIV | PB2 | 1027 | G | A | 1 | E343K | Non_Synonymous | PB2 cap-binding region with m^7^GTP (Guilligay et al., 2008) | Viral genome transportation, transcription and replication |
| 4481 | SINGLE LAIV | PB2 | 1079 | A | G | 1.1 | Y360C | Non_Synonymous | PB2 cap-binding region with m^7^GTP (Guilligay et al., 2008) | Viral genome transportation, transcription and replication |
| 4481 | SINGLE LAIV | PB2 | 1088 | T | C | 1 | F363S | Non_Synonymous | PB2 cap-binding region with m^7^GTP (Guilligay et al., 2008) | Viral genome transportation, transcription and replication |
| 4486 | PRIME BOOST | PB2 | 29 | T | A | 25.4 | L10Q | Non_Synonymous | PB2 (N-terminal) – PB1 (C-terminal) binding region (Sugiyama et al., 2009) | Viral genome/protein interaction |
| 4486 | PRIME BOOST | PB2 | 82 | A | G | 1.2 | M28V | Non_Synonymous | PB2 (N-terminal) – PB1 (C-terminal) binding region (Sugiyama et al., 2009) | Viral genome/protein interaction |
| 4486 | PRIME BOOST | PB2 | 1205 | T | C | 1.3 | M402T | Non_Synonymous | PB2 cap-binding region with m^7^GTP (Guilligay et al., 2008) | Viral genome transportation, transcription and replication |
| 4486 | PRIME BOOST | PB2 | 1483 | G | A | 4.9 | V495I | Non_Synonymous | PB2 nuclear localization region (Mukaigawa & Nayak, 1991) | Viral genome transportation, transcription and replication |
| 4486 | PRIME BOOST | PB2 | 2257 | A | G | 1.8 | R753G | Non_Synonymous | PB2 nuclear localization region (Tarendeau et al., 2007) | Viral genome transportation, transcription and replication |
| 4490 | NO VAC | PB2 | 1396 | G | A | 16.8 | D466N | Non_Synonymous | PB2 nuclear localization region and cap-binding region with m^7^GTP (Guilligay et al., 2008; Mukaigawa & Nayak, 1991) | Viral genome transportation, transcription and replication |
| 4499 | PRIME BOOST | PB2 | 1273 | A | G | 1.5 | N425D | Non_Synonymous | PB2 cap-binding region with m^7^GTP (Guilligay et al., 2008) | Viral genome transportation, transcription and replication |
| 4499 | PRIME BOOST | PB2 | 2221 | T | C | 1.8 | S741P | Non_Synonymous | PB2 nuclear localization region (Mukaigawa & Nayak, 1991) | Viral genome transportation, transcription and replication |
| 4552 | PRIME BOOST | PB2 | 766 | G | A | 1.6 | D256N | Non_Synonymous | Associated with the influenza polymerase activity of the RNP expressed (Manzoor et al., 2009) | Viral genome transportation, transcription and replication |
| 4945 | SINGLE LAIV | PB2 | 1062 | A | G | 2.1 | I354M | Non_Synonymous | PB2 cap-binding region with m^7^GTP (Guilligay et al., 2008) | Viral genome transportation, transcription and replication |
| 5174 | NO VAC | PB2 | 2218 | G | T | 1.2 | D740Y | Non_Synonymous | PB2 nuclear localization region (Mukaigawa & Nayak, 1991) | Viral genome transportation, transcription and replication |
| 5185 | PRIME BOOST | PB2 | 1106 | G | A | 1.6 | R369K | Non_Synonymous | PB2 cap-binding region with m^7^GTP (Guilligay et al., 2008) | Viral genome transportation, transcription and replication |

**Reference**

Air, G. M. (1979). Nucleotide sequence coding for the “signal peptide” and N terminus of the hemagglutinin from an Asian (H2N2) strain of influenza virus. *Virology*, *97*(2), 468–472.

Albo, C., Valencia, A., & Portela, A. (1995). Identification of an RNA binding region within the N-terminal third of the influenza A virus nucleoprotein. *Journal of Virology*, *69*(6), 3799–3806.

Aragón, T., de la Luna, S., Novoa, I., Carrasco, L., Ortín, J., & Nieto, A. (2000). Eukaryotic translation initiation factor 4GI is a cellular target for NS1 protein, a translational activator of influenza virus. *Molecular and Cellular Biology*, *20*(17), 6259–6268.

Baudin, F., Petit, I., Weissenhorn, W., & Ruigrok, R. W. H. (2001). In vitro dissection of the membrane and RNP binding activities of influenza virus M1 protein. *Virology*, *281*(1), 102–108.

Biswas, S. K., Boutz, P. L., & Nayak, D. P. (1998). Influenza virus nucleoprotein interacts with influenza virus polymerase proteins. *Journal of Virology*, *72*(7), 5493–5501.

Bornholdt, Z. A., & Prasad, B. V. V. (2006). X-ray structure of influenza virus NS1 effector domain. *Nature Structural & Molecular Biology*, *13*(6), 559–560.

Bornholdt, Z. A., & Prasad, B. V. V. (2008). X-ray structure of NS1 from a highly pathogenic H5N1 influenza virus. *Nature*, *456*(7224), 985–988.

Cheng, A., Wong, S. M., & Yuan, Y. A. (2009). Structural basis for dsRNA recognition by NS1 protein of influenza A virus. *Cell Research*, *19*(2), 187–195.

Das, K., Ma, L.-C., Xiao, R., Radvansky, B., Aramini, J., Zhao, L., … Arnold, E. (2008). Structural basis for suppression of a host antiviral response by influenza A virus. *Proceedings of the National Academy of Sciences*, *105*(35), 13093–13098.

Desmet, E. A., Bussey, K. A., Stone, R., & Takimoto, T. (2013). Identification of the N-terminal domain of the influenza virus PA responsible for the suppression of host protein synthesis. *Journal of Virology*, *87*(6), 3108–3118.

Ehrhardt, C., Wolff, T., Pleschka, S., Planz, O., Beermann, W., Bode, J. G., … Ludwig, S. (2007). Influenza A virus NS1 protein activates the PI3K/Akt pathway to mediate antiapoptotic signaling responses. *Journal of Virology*, *81*(7), 3058–3067.

Elton, D., Medcalf, E., Bishop, K., & Digard, P. (1999). Oligomerization of the influenza virus nucleoprotein: identification of positive and negative sequence elements. *Virology*, *260*(1), 190–200.

Elton, D., Medcalf, L., Bishop, K., Harrison, D., & Digard, P. (1999). Identification of amino acid residues of influenza virus nucleoprotein essential for RNA binding. *Journal of Virology*, *73*(9), 7357–7367.

Gallacher, M., Brown, S. G., Hale, B. G., Fearns, R., Olver, R. E., Randall, R. E., & Wilson, S. M. (2009). Cation currents in human airway epithelial cells induced by infection with influenza A virus. *The Journal of Physiology*, *587*(13), 3159–3173.

Grantham, M. L., Wu, W.-H., Lalime, E. N., Lorenzo, M. E., Klein, S. L., & Pekosz, A. (2009). Palmitoylation of the influenza A virus M2 protein is not required for virus replication in vitro but contributes to virus virulence. *Journal of Virology*, *83*(17), 8655–8661.

Gregoriades, A., & Frangione, B. (1981). Insertion of influenza M protein into the viral lipid bilayer and localization of site of insertion. *Journal of Virology*, *40*(1), 323–328.

Guilligay, D., Tarendeau, F., Resa-Infante, P., Coloma, R., Crepin, T., Sehr, P., … Hart, D. J. (2008). The structural basis for cap binding by influenza virus polymerase subunit PB2. *Nature Structural & Molecular Biology*, *15*(5), 500.

Hale, B. G., Barclay, W. S., Randall, R. E., & Russell, R. J. (2008). Structure of an avian influenza A virus NS1 protein effector domain. *Virology*, *378*(1), 1–5.

Hale, B. G., Jackson, D., Chen, Y.-H., Lamb, R. A., & Randall, R. E. (2006). Influenza A virus NS1 protein binds p85β and activates phosphatidylinositol-3-kinase signaling. *Proceedings of the National Academy of Sciences*, *103*(38), 14194–14199.

Hale, B. G., Kerry, P. S., Jackson, D., Precious, B. L., Gray, A., Killip, M. J., … Russell, R. J. (2010). Structural insights into phosphoinositide 3-kinase activation by the influenza A virus NS1 protein. *Proceedings of the National Academy of Sciences*, *107*(5), 1954–1959.

Hale, B. G., Knebel, A., Botting, C. H., Galloway, C. S., Precious, B. L., Jackson, D., … Randall, R. E. (2009). CDK/ERK-mediated phosphorylation of the human influenza A virus NS1 protein at threonine-215. *Virology*, *383*(1), 6–11.

Hale, B. G., Randall, R. E., Ortín, J., & Jackson, D. (2008). The multifunctional NS1 protein of influenza A viruses. *Journal of General Virology*, *89*(10), 2359–2376.

Hale, B. G., Steel, J., Medina, R. A., Manicassamy, B., Ye, J., Hickman, D., … Perez, D. R. (2010). Inefficient control of host gene expression by the 2009 pandemic H1N1 influenza A virus NS1 protein. *Journal of Virology*, *84*(14), 6909–6922.

He, X., Zhou, J., Bartlam, M., Zhang, R., Ma, J., Lou, Z., … Zeng, Z. (2008). Crystal structure of the polymerase PA C–PB1 N complex from an avian influenza H5N1 virus. *Nature*, *454*(7208), 1123–1126.

Hui, E. K.-W., Smee, D. F., Wong, M.-H., & Nayak, D. P. (2006). Mutations in influenza virus M1 CCHH, the putative zinc finger motif, cause attenuation in mice and protect mice against lethal influenza virus infection. *Journal of Virology*, *80*(12), 5697–5707.

Jung, T. E., & Brownlee, G. G. (2006). A new promoter-binding site in the PB1 subunit of the influenza A virus polymerase. *Journal of General Virology*, *87*(3), 679–688.

Kochs, G., García-Sastre, A., & Martínez-Sobrido, L. (2007). Multiple anti-interferon actions of the influenza A virus NS1 protein. *Journal of Virology*, *81*(13), 7011–7021.

Li, Y., Yamakita, Y., & Krug, R. M. (1998). Regulation of a nuclear export signal by an adjacent inhibitory sequence: the effector domain of the influenza virus NS1 protein. *Proceedings of the National Academy of Sciences*, *95*(9), 4864–4869.

Long, J.-X., Peng, D.-X., Liu, Y.-L., Wu, Y.-T., & Liu, X.-F. (2008). Virulence of H5N1 avian influenza virus enhanced by a 15-nucleotide deletion in the viral nonstructural gene. *Virus Genes*, *36*(3), 471–478.

Manzoor, R., Sakoda, Y., Nomura, N., Tsuda, Y., Ozaki, H., Okamatsu, M., & Kida, H. (2009). PB2 protein of a highly pathogenic avian influenza virus strain A/chicken/Yamaguchi/7/2004 (H5N1) determines its replication potential in pigs. *Journal of Virology*, *83*(4), 1572–1578.

Mukaigawa, J., & Nayak, D. P. (1991). Two signals mediate nuclear localization of influenza virus (A/WSN/33) polymerase basic protein 2. *Journal of Virology*, *65*(1), 245–253.

Noah, D. L., Twu, K. Y., & Krug, R. M. (2003). Cellular antiviral responses against influenza A virus are countered at the posttranscriptional level by the viral NS1A protein via its binding to a cellular protein required for the 3′ end processing of cellular pre-mRNAS. *Virology*, *307*(2), 386–395.

Poole, E. L., Medcalf, L., Elton, D., & Digard, P. (2007). Evidence that the C-terminal PB2-binding region of the influenza A virus PB1 protein is a discrete α-helical domain. *FEBS Letters*, *581*(27), 5300–5306.

Schroeder, C., Heider, H., Möncke-Buchner, E., & Lin, T.-I. (2005). The influenza virus ion channel and maturation cofactor M2 is a cholesterol-binding protein. *European Biophysics Journal*, *34*(1), 52–66.

Seo, S. H., Hoffmann, E., & Webster, R. G. (2002). Lethal H5N1 influenza viruses escape host anti-viral cytokine responses. *Nature Medicine*, *8*(9), 950–954.

Shimizu, T., Takizawa, N., Watanabe, K., Nagata, K., & Kobayashi, N. (2011). Crucial role of the influenza virus NS2 (NEP) C-terminal domain in M1 binding and nuclear export of vRNP. *FEBS Letters*, *585*(1), 41–46.

Shin, Y.-K., Li, Y., Liu, Q., Anderson, D. H., Babiuk, L. A., & Zhou, Y. (2007). SH3 binding motif 1 in influenza A virus NS1 protein is essential for PI3K/Akt signaling pathway activation. *Journal of Virology*, *81*(23), 12730–12739.

Sugiyama, K., Obayashi, E., Kawaguchi, A., Suzuki, Y., Tame, J. R. H., Nagata, K., & Park, S. (2009). Structural insight into the essential PB1–PB2 subunit contact of the influenza virus RNA polymerase. *The EMBO Journal*, *28*(12), 1803–1811.

Tarendeau, F., Boudet, J., Guilligay, D., Mas, P. J., Bougault, C. M., Boulo, S., … Ellenberg, J. (2007). Structure and nuclear import function of the C-terminal domain of influenza virus polymerase PB2 subunit. *Nature Structural & Molecular Biology*, *14*(3), 229–233.

Toyoda, T., Adyshev, D. M., Kobayashi, M., Iwata, A., & Ishihama, A. (1996). Molecular assembly of the influenza virus RNA polymerase: determination of the subunit-subunit contact sites. *Journal of General Virology*, *77*(9), 2149–2157.

Twu, K. Y., Kuo, R.-L., Marklund, J., & Krug, R. M. (2007). The H5N1 influenza virus NS genes selected after 1998 enhance virus replication in mammalian cells. *Journal of Virology*, *81*(15), 8112–8121.

Weber, F., Kochs, G., Gruber, S., & Haller, O. (1998). A classical bipartite nuclear localization signal on Thogoto and influenza A virus nucleoproteins. *Virology*, *250*(1), 9–18.

Ye, Q., Krug, R. M., & Tao, Y. J. (2006). The mechanism by which influenza A virus nucleoprotein forms oligomers and binds RNA. *Nature*, *444*(7122), 1078–1082.

Ye, Z., Liu, T., Offringa, D. P., McInnis, J., & Levandowski, R. A. (1999). Association of influenza virus matrix protein with ribonucleoproteins. *Journal of Virology*, *73*(9), 7467–7473.

Yin, C., Khan, J. A., Swapna, G. V. T., Ertekin, A., Krug, R. M., Tong, L., & Montelione, G. T. (2007). Conserved surface features form the double-stranded RNA binding site of non-structural protein 1 (NS1) from influenza A and B viruses. *Journal of Biological Chemistry*, *282*(28), 20584–20592.
