# Supplementary material for: Vaccination decreases the risk of influenza A virus reassortment but not genetic variation in pigs
Source: eLife. 2022 Sep 2;11:e78618. doi: 10.7554/eLife.78618 (PMC9439680; doi:10.7554/eLife.78618)
Supplement: Supplementary file 5. [file elife-78618-supp5.docx]

**Supplementary file 5. Spearman correlation between humoral and cellular immune responses and IAV HA nucleotide diversity in individual pigs.**

| **Virus** | **Sample size (n)** | **Variable 1** | **Variable 2^c^** | **R^d^** | **P value** |
| --- | --- | --- | --- | --- | --- |
| H1N1 | 17 | H1-specific hemagglutinin inhibition titer^a^ | Pi | 0.129 | 0.622 |
|  |  |  | PiN | 0.138 | 0.598 |
|  |  |  | PiS | 0.138 | 0.597 |
|  | 13 | H1-specific IFN-γ ELISPOT cell count^b^ | Pi | 0.087 | 0.778 |
|  |  |  | PiN | 0.110 | 0.720 |
|  |  |  | PiS | -0.074 | 0.811 |
| H3N2 | 20 | H3-specific hemagglutinin inhibition titer | Pi | -0.203 | 0.391 |
|  |  |  | PiN | 0.205 | 0.385 |
|  |  |  | PiS | -0.111 | 0.641 |
|  | 16 | H3-specific IFN-γ ELISPOT cell count | Pi | -0.031 | 0.909 |
|  |  |  | PiN | -0.512 | 0.043 |
|  |  |  | PiS | 0.225 | 0.402 |

^a^ The hemagglutinin inhibition assay was performed on blood samples from selected treatment pigs collected prior to contact with challenged pigs.

^b^ The analysis of ELISPOT on IFN-γ secreting cells was performed on lymph nodes from selected treatment pigs which were collected at necropsy 7 days post contact with seeder pigs.

^c^ All the parameters of nucleotide diversity were calculated in reference to the HA segment of the H1 or H3 viruses.

^d^ Abbreviations: R: Spearman's rank correlation coefficient; Pi: overall nucleotide diversity; PiN: nonsynonymous nucleotide diversity; PiS: synonymous nucleotide diversity.
