# Supplementary material for: Vaccination decreases the risk of influenza A virus reassortment but not genetic variation in pigs
Source: eLife. 2022 Sep 2;11:e78618. doi: 10.7554/eLife.78618 (PMC9439680; doi:10.7554/eLife.78618)
Supplement: Supplementary file 6. [file elife-78618-supp6.docx]

**Supplementary file 6. Clade classification and homology of each gene segment between A/swine/Minnesota/PAH-618/2011 (H1N1) and A/swine/Minnesota/080470/2015 (H3N2) viruses.**

| **Gene Segment** | **Clade^a^** | | **% Nucleotide Homology** | **Protein** | **% Amino Acid Homology** |
| --- | --- | --- | --- | --- | --- |
|  | **H1N1** | **H3N2** |  |  |  |
| PB2 | TRIG | TRIG | 95.88% | PB2 | 98.42% |
| PB1 | TRIG | TRIG | 98.33% | PB1 | 99.34% |
|  |  |  |  | PB1-F2 | 96.20% |
| PA | H1N1pdm09 | TRIG | 92.14% | PA | 96.51% |
|  |  |  |  | PA-X | 94.40% |
| HA | Gamma  1A3.3.3 | 2010 human like 3.2010.1 | 52.16% | HA | 41.70% |
| NP | H1N1pdm09 | TRIG | 93.05% | NP | 96.79% |
| NA | Classical swine | N2-2002A | 53.56% | NA | 42.17% |
| M | H1N1pdm09 | H1N1pdm09 | 97.76% | M1 | 99.21% |
|  |  |  |  | M2 | 92.78% |
| NS | TRIG | TRIG | 97.61% | NS1 | 95.89% |
|  |  |  |  | NS2 | 100.00% |

^a^ The clade classifications of each gene segment was determined by automated classification tool OctoFLU (https://github.com/flu-crew/octoFLU).
