# Supplementary material for: Vaccination decreases the risk of influenza A virus reassortment but not genetic variation in pigs
Source: eLife. 2022 Sep 2;11:e78618. doi: 10.7554/eLife.78618 (PMC9439680; doi:10.7554/eLife.78618)
Supplement: MDAR checklist [file elife-78618-mdarchecklist1.docx]

**Materials Design Analysis Reporting (MDAR)**

**Checklist for Authors**

The [MDAR framework](https://osf.io/xfpn4/) establishes a minimum set of requirements in transparent reporting mainly applicable to studies in the life sciences.

*eLife* asks authors to **provide detailed information within their article** to facilitate the interpretation and replication of their work. Authors can also upload supporting materials to comply with relevant reporting guidelines for health-related research (see [EQUATOR Network](http://www.equator-network.org/%20)), life science research (see the [BioSharing Information Resource](http://biosharing.org/)), or animal research (see the [ARRIVE Guidelines](http://www.plosbiology.org/article/info:doi/10.1371/journal.pbio.1000412) and the [STRANGE Framework](https://doi.org/10.1038/d41586-020-01751-5); for details, see *eLife*’s [Journal Policies](https://reviewer.elifesciences.org/author-guide/journal-policies)). Where applicable, authors should refer to any relevant reporting standards materials in this form.

For all that apply, please note **where in the article** the information is provided. Please note that we also collect information about data availability and ethics in the submission form.

**Materials:**

| **Newly created materials** | **Indicate where provided: section/figure legend** | **N/A** |
| --- | --- | --- |
| The manuscript includes a dedicated "materials availability statement" providing transparent disclosure about availability of newly created materials including details on how materials can be accessed and describing any restrictions on access. | Not particularly applicable for this study. Only the isolated influenza plaques could be considered as new created materials which stored in -80 ℃ freezer in Torremorell’s lab (Materials and Methods – Plaque library preparation). | N/A |
|  |  |  |
| **Antibodies** | **Indicate where provided: section/figure legend** | **N/A** |
| For commercial reagents, provide supplier name, catalogue number and [RRID](https://scicrunch.org/resources), if available. | No antibodies used in this study. | N/A |
|  |  |  |
| **DNA and RNA sequences** | **Indicate where provided: section/figure legend** | **N/A** |
| Short novel DNA or RNA including primers, probes: Sequences should be included or deposited in a public repository. | All the raw sequence reads from the BALF samples and influenza plaques are deposited in Bioproject PRJNA813974 (Materials and Methods – Data availability). |  |
|  |  |  |
| **Cell materials** | **Indicate where provided: section/figure legend** | **N/A** |
| Cell lines: Provide species information, strain. Provide accession number in repository OR supplier name, catalog number, clone number, OR RRID. | The Madin-Darby Canine Kidney (MDCK) cells used in this study is obtained from the University of Minnesota Veterinary Diagnostic Laboratory (VDL) (Materials and Methods – Plaque library preparation). |  |
| Primary cultures: Provide species, strain, sex of origin, genetic modification status. | No primary cell cultures in this study. | N/A |
|  |  |  |
| **Experimental animals** | **Indicate where provided: section/figure legend** | **N/A** |
| Laboratory animals or Model organisms: Provide species, strain, sex, age, genetic modification status. Provide accession number in repository OR supplier name, catalog number, clone number, OR RRID. | No laboratory animals used in this study and all the samples analyzed in this study were collected from pigs from a previously published vaccination-challenge research. The detailed information of sampled pigs can be found in cited reference in Materials and Methods – vaccine-challenge experiment in pigs. |  |
| Animal observed in or captured from the field: Provide species, sex, and age where possible. | Cross-bred pigs of weaning age (~25 days of age). Detailed information was cited in an associated reference in Materials and Methods – vaccine-challenge experiment in pigs. |  |
|  |  |  |
| **Plants and microbes** | **Indicate where provided: section/figure legend** | **N/A** |
| Plants: provide species and strain, ecotype and cultivar where relevant, unique accession number if available, and source (including location for collected wild specimens). | No plant materials used in this study. | N/A |
| Microbes: provide species and strain, unique accession number if available, and source. | The source and gene sequences of two challenge influenza strains (H1N1 and H3N2) in this study can be found in GenBank with accession numbers MT377710 – MT377725 (Materials and Methods – vaccine-challenge experiment in pigs). |  |
|  |  |  |
| **Human research participants** | **Indicate where provided: section/figure legend) or state if these demographics were not collected** | **N/A** |
| If collected and within the bounds of privacy constraints report on age, sex, gender and ethnicity for all study participants. | No human research participants in this study. | N/A |

**Design:**

| **Study protocol** | **Indicate where provided: section/figure legend** | **N/A** |
| --- | --- | --- |
| If the study protocol has been pre-registered, provide DOI. For clinical trials, provide the trial registration number OR cite DOI. | Not applicable to our study. | N/A |
|  |  |  |
| **Laboratory protocol** | **Indicate where provided: section/figure legend** | **N/A** |
| Provide DOI OR other citation details if detailed step-by-step protocols are available. | The associated citations of laboratory protocols can be found throughout the section of Materials and Methods. |  |
|  |  |  |
| **Experimental study design (statistics details) *** | | |
| **For in vivo studies: State whether and how the following have been done** | **Indicate where provided: section/figure legend. If it could have been done, but was not, write “not done”** | **N/A** |
| Sample size determination | No explicit power analysis was performed. We used all the available samples from a vaccination-challenge study. | N/A |
| Randomisation | The randomization of pig assignment and influenza plaques picked up were described in associated reference and the Materials and Methods – vaccine-challenge experiment in pigs and Plaque library preparation. |  |
| Blinding | Blinding is not applicable in this study. | N/A |
| Inclusion/exclusion criteria | The detailed inclusion/exclusion criteria of sample selection for direct sequencing and plaque purification, and sequence analysis for influenza reassortant and single nucleotide variant identification have been illustrated in Materials and Methods. |  |
|  |  |  |
| **Sample definition and in-laboratory replication** | **Indicate where provided: section/figure legend** | **N/A** |
| State number of times the experiment was replicated in the laboratory. | Experiment was not replicated in the laboratory because it was based out of samples collected from experimentally infected animals and that all the samples were used. | N/A |
| Define whether data describe technical or biological replicates. | Not applicable to our study. | N/A |
|  |  |  |
| **Ethics** | **Indicate where provided: section/submission form** | **N/A** |
| Studies involving human participants: State details of authority granting ethics approval (IRB or equivalent committee(s), provide reference number for approval. | No human participants involved. | N/A |
| Studies involving experimental animals: State details of authority granting ethics approval (IRB or equivalent committee(s), provide reference number for approval. | The animal works where the samples of this study collected from were approved by the IACUC (Protocol ID: 1712-35407A) and IBC (Protocol ID: 1508-32918H) committees from the University of Minnesota (Materials and Methods – vaccine-challenge experiment in pigs). |  |
| Studies involving specimen and field samples: State if relevant permits obtained, provide details of authority approving study; if none were required, explain why. | No field samples or specimen involved in this study. | N/A |
|  |  |  |
| **Dual Use Research of Concern (DURC)** | **Indicate where provided: section/submission form** | **N/A** |
| If study is subject to dual use research of concern regulations, state the authority granting approval and reference number for the regulatory approval. | Our study is not subject to dual use research of concern. | N/A |

**Analysis:**

| **Attrition** | **Indicate where provided: section/figure legend** | **N/A** |
| --- | --- | --- |
| Describe whether exclusion criteria were pre-established. Report if sample or data points were omitted from analysis. If yes, report if this was due to attrition or intentional exclusion and provide justification. | The exclusion criteria of samples used for direct sequencing and plaque purification, influenza single nucleotide variant identification, and the sequences applied for recognizing the influenza reassortant were pre-established and described throughout Materials and Methods. No data points were omitted during the further evolutionary analysis and data visualization. |  |
|  |  |  |
| **Statistics** | **Indicate where provided: section/figure legend** | **N/A** |
| Describe statistical tests used and justify choice of tests. | The statistical tests performed in this study were illustrated in figure legends and Materials and Methods – Statistical analysis. |  |
|  |  |  |
| **Data availability** | **Indicate where provided: section/submission form** | **N/A** |
| For newly created and reused datasets, the manuscript includes a data availability statement that provides details for access (or notes restrictions on access). | The data availability statement can be found in Materials and Methods – Data availability. |  |
| When newly created datasets are publicly available, provide accession number in repository OR DOI and licensing details where available. | The raw datasets created in this study have been deposited in https://github.com/TorremorellLabUMN/Swine-IAV-within-host-evolution (Materials and Methods – Data availability). |  |
| If reused data is publicly available provide accession number in repository OR DOI, OR URL, OR citation. | The genome sequences of two challenge influenza strains (H1N1 and H3N2) in this study were deposited in GenBank with accession numbers MT377710 – MT377725. The IFN-γ ELISPOT counts and HI titers from the pigs were previously measured in published research which were cited in Materials and Methods – vaccine-challenge experiment in pigs. |  |
|  |  |  |
| **Code availability** | **Indicate where provided: section/figure legend** | **N/A** |
| For any computer code/software/mathematical algorithms essential for replicating the main findings of the study, whether newly generated or re-used, the manuscript includes a data availability statement that provides details for access or notes restrictions. | All the software used in this study have been cited throughout the Materials and Methods. The data availability statement can be found in Materials and Methods – Data availability. |  |
| Where newly generated code is publicly available, provide accession number in repository, OR DOI OR URL and licensing details where available. State any restrictions on code availability or accessibility. | All the codes generated in this study have been deposited in https://github.com/TorremorellLabUMN/Swine-IAV-within-host-evolution (Materials and Methods – Data availability). |  |
| If reused code is publicly available provide accession number in repository OR DOI OR URL, OR citation. | No codes reused in this study. | N/A |

**Reporting:**

The MDAR framework recommends adoption of discipline-specific guidelines, established and endorsed through community initiatives.

| **Adherence to community standards** | **Indicate where provided: section/figure legend** | **N/A** |
| --- | --- | --- |
| State if relevant guidelines (e.g., ICMJE, MIBBI, ARRIVE, STRANGE) have been followed, and whether a checklist (e.g., CONSORT, PRISMA, ARRIVE) is provided with the manuscript. | The manuscript preparations are followed by ICMJE guidelines. |  |

* We provide the following guidance regarding transparent reporting and statistics; we also refer authors to [Ten common statistical mistakes to watch out for when writing or reviewing a manuscript](https://doi.org/10.7554/eLife.48175).

**Sample-size estimation**

- You should state whether an appropriate sample size was computed when the study was being designed
- You should state the statistical method of sample size computation and any required assumptions
- If no explicit power analysis was used, you should describe how you decided what sample (replicate) size (number) to use

**Replicates**

- You should report how often each experiment was performed
- You should include a definition of biological versus technical replication
- The data obtained should be provided and sufficient information should be provided to indicate the number of independent biological and/or technical replicates
- If you encountered any outliers, you should describe how these were handled
- Criteria for exclusion/inclusion of data should be clearly stated
- High-throughput sequence data should be uploaded before submission, with a private link for reviewers provided (these are available from both GEO and ArrayExpress)

**Statistical reporting**

- Statistical analysis methods should be described and justified
- Raw data should be presented in figures whenever informative to do so (typically when N per group is less than 10)
- For each experiment, you should identify the statistical tests used, exact values of N, definitions of center, methods of multiple test correction, and dispersion and precision measures (e.g., mean, median, SD, SEM, confidence intervals; and, for the major substantive results, a measure of effect size (e.g., Pearson's r, Cohen's d)
- Report exact p-values wherever possible alongside the summary statistics and 95% confidence intervals. These should be reported for all key questions and not only when the p-value is less than 0.05.

**Group allocation**

- Indicate how samples were allocated into experimental groups (in the case of clinical studies, please specify allocation to treatment method); if randomization was used, please also state if restricted randomization was applied
- Indicate if masking was used during group allocation, data collection and/or data analysis
